# ﻿Revision of the Neotropical species of the hoverfly genus *Serichlamys* Curran, 1925 (Diptera, Syrphidae, Microdontinae)

**DOI:** 10.3897/zookeys.1243.132809

**Published:** 2025-06-24

**Authors:** Menno Reemer, Ximo Mengual

**Affiliations:** 1 Naturalis Biodiversity Center, P.O. Box 9517, 2300 RA Leiden, Netherlands Naturalis Biodiversity Center Leiden Netherlands; 2 Museum Koenig Bonn, Leibniz-Institut zur Analyse des Biodiversitätswandels, Adenauerallee 127, 53113 Bonn, Germany Museum Koenig Bonn Bonn Germany; 3 Instituto Nacional de Biodiversidad, División de Entomología, Quito, Ecuador Instituto Nacional de Biodiversidad Quito Ecuador

**Keywords:** Ant flies, Central America, COI barcodes, distribution, identification key, morphology, new species, South America, taxonomy

## Abstract

The Neotropical species of the hoverfly genus *Serichlamys* Curran, 1925 are revised. A total number of 14 Neotropical species are recognized, two of which were previously described, namely *S.mitis* (Curran, 1940) and *S.mus* (Curran, 1936). The other 12 species are here described for the first time: *S.boti* Reemer, **sp. nov.**, *S.chloraspis* Reemer, **sp. nov.**, *S.melamitis* Reemer, **sp. nov.**, *S.mellimitis* Reemer, **sp. nov.**, *S.pallitarsis* Reemer & Mengual, **sp. nov.**, *S.serpentiphallus* Reemer, **sp. nov.**, *S.simpliciphallus* Reemer, **sp. nov.**, *S.spathulata* Reemer, **sp. nov.**, *S.trigonoides* Reemer, **sp. nov.**, *S.varicaudata* Reemer & Mengual, **sp. nov.**, *S.vexilliphallus* Reemer & Mengual, **sp. nov.**, and *S.xanthocnemia* Reemer, **sp. nov.** An identification key is provided including all the recognised species. The distribution of the genus in the Neotropical region is shown to be disjunct, with one group of species in the northwest of the South American landmass and another group in southeastern Brazil.

## ﻿Introduction

In the current taxonomic concept of the genus, *Serichlamys* Curran, 1925 comprises moderately-sized (5.5–12 mm), mostly dark coloured hoverflies with a more or less oval abdomen. Nothing is known about the larval biology of the *Serichlamys* species, although the larvae are likely to be associated with ants, as are all other Microdontinae for which the larvae are known (Reemer 2013a). *Serichlamys* was originally erected as a subgenus of *Microdon* Meigen, 1803 by Curran (1925), with the Nearctic taxon *Aphritisrufipes* Macquart, 1842 as type species. Curran did not state which characters he considered diagnostic for his subgenus, but in his key to the species of *Microdon*, *M.rufipes* was keyed out based on the eyes being pilose. Curran apparently had not seen specimens of this species himself, as he only cites the English translation that Williston (1886) gave of the original French description by Macquart (1842). Macquart had stated ‘yeux peu velus’ [eyes little pilose], which was translated by Williston as ‘eyes thinly pilose’, after which Curran stated ‘eyes pilose’. Examination of the type specimen of *A.rufipes* by Reemer and Ståhls (2013a), as well as subsequent examination of additional specimens by the first author, revealed that the eyes of this species are actually bare. *Serichlamys* Curran was considered a subjective synonym of *Microdon* Meigen by Wirth et al. (1965), an opinion followed by Thompson (1981) and Cheng and Thompson (2008). Reemer and Ståhls (2013a, b) reinstated *Serichlamys* as a valid genus, based on an analysis of combined molecular and morphological data. The taxon was recovered as sister group to the Old World genus *Archimicrodon* Hull, 1945. Preliminary results based on an extensive molecular dataset (unpublished) confirm this relationship. These unpublished results also confirm the sister relationship of *Microdonrufipes* Macquart, the type species of the genus *Serichlamys*, with one of the Neotropical species included in *Serichlamys*. The genus can be identified using the keys of Reemer and Ståhls (2013a) and Reemer (2016).

The present work revises the 14 Neotropical species of *Serichlamys*. Two of them, i.e., *S.mitis* (Curran, 1940) and *S.mus* (Curran, 1936), were previously described and are included in a key to South American Microdontinae by Curran (1941). The other 12 species are here described for the first time. In addition, an identification key is provided to all the Neotropical species of the genus.

## ﻿Materials and methods

### ﻿Terminology, holding institutions, and figures

Morphological terminology largely follows Cumming and Wood (2017) (for wing venation the ‘traditional’ system by McAlpine (1981) is used), supplemented with some terms specifically introduced for Microdontinae by Reemer and Ståhls (2013b). Ratios of lengths of antennal segments are notated as scape:pedicel:postpedicel.

Type material of previously described species has been studied when available to us. For studied primary types, text on labels is given ad verbatim. Text is indicated in quotation marks (“ ”) and each line on the label is separated by a double forward slash (//). Text not given on labels (i.e., remarks by authors) is given in square brackets ([]).

The following acronyms are used to indicate institutional and private collections:

**AMNH**American Museum of Natural History, New York (USA);

**CEUA**Colección Entomológica de la Universidad de Antioquia, Medellín (Colombia);

**CNC**Canadian National Collection of Insects, Arachnids and Nematodes, Ottawa (Canada);

**CSCA**California State Collection of Arthropods, Sacramento (USA);

**DEBU**University of Guelph Insect Collection, Guelph (Canada);

**INABIO** Instituto Nacional de Biodiversidad, Quito (Ecuador);

**RBINS**Institut Royal des Sciences Naturelles de Belgique, Brussels (Belgium);

**JTS** John T. Smit, Utrecht (the Netherlands);

**MNCR**Museo Nacional de Costa Rica, San José (Costa Rica);

**MZH**Finnish Museum of Natural History, Helsinki (Finland);

**MZUSP**Museu de Zoologia da Universidade de São Paulo, São Paulo (Brazil);

**NHMUK**Natural History Museum, London (United Kingdom);

**RMNH**Naturalis Biodiversity Center, Leiden (the Netherlands);

**UCRC**University of California, Dep. of Entomology, Riverside (USA);

**USNM**National Museum of Natural History, Washington DC (USA);

**ZMUC**Zoological Museum University of Copenhagen, Copenhagen (Denmark);

**ZFMK**Museum Koenig Bonn, Leibniz-Institut zur Analyse des Biodiversitätswandels, Bonn (Germany).

Photos were taken using a motorised Zeiss Discovery V20 stereomicroscope, combined with Zeiss ZEN 3.8 image stacking software. Male genitalia were dissected and macerated in lactic acid at room temperature for 24 hours, after which they were rinsed in distilled water and stored in glycerol. Drawings of the genitalia were made based on photos of these preparations. Distribution maps were made using QGIS v. 3.22 and Adobe Illustrator.

### ﻿DNA barcoding

For selected *Serichlamys* specimens, the 5′-end of the mitochondrial cytochrome oxidase *c* subunit I (COI) gene was sequenced. One or two legs were used for DNA extraction and the remainder of the individual was kept for morphological comparison and properly labelled as DNA voucher. DNA was extracted following standard protocols of the commercially available DNeasy Blood & Tissue Kit (QIAgen®). The COI barcode region was amplified and sequenced using the forward primer LCO1-1490 and the reverse primer COI-Dipt-2183R following the protocol by Mengual et al. (2022) for specimens sequenced at the ZFMK. PCR amplification, purification, sequencing protocols, and editing were carried out as described in Rozo-Lopez and Mengual (2015) and Mengual et al. (2022) for specimens sequenced at the ZFMK. DNA primers, as well as amplification, purification, sequencing protocols, and edition were carried out as described in Gibson et al. (2010) for specimens sequenced at CNC. GenBank accession numbers are listed for each sequenced specimen in the Examined material section.

### ﻿Molecular analysis

Public sequences of *Serichlamys* species available at BOLD (https://www.boldsystems.org/index.php; accessed on 8 July 2024) were downloaded. Together with the newly obtained sequences, an alignment of the COI sequences without gaps or stop codons using Geneious Prime 2022.1.1 (Biomatters Ltd) was produced. DNA sequences can be accessed via the dataset DS-SERICHLA in BOLD (http://dx.doi.org/10.5883/DS-SERICHLA) and in GenBank (https://www.ncbi.nlm.nih.gov/genbank/). A distance-based Neighbour-Joining (NJ) analysis was done using the Jukes-Cantor Model as implemented in the software Geneious Prime 2022.1.1. The DNA barcode of *Aristosyrphuscarpenteri* (Hull, 1945) (GenBank accession number ON943475) was constrained as the root for the NJ tree. Bootstrap support values (BS) were estimated from 1000 replicates directly from Geneious Prime. The NJ tree was drawn with the aid of FigTree v. 1.3.1 (Rambaut 2018) and Adobe Illustrator CS 5.1.

## ﻿Results

### ﻿Differential diagnosis of *Serichlamys*

**Figures 1–4.**
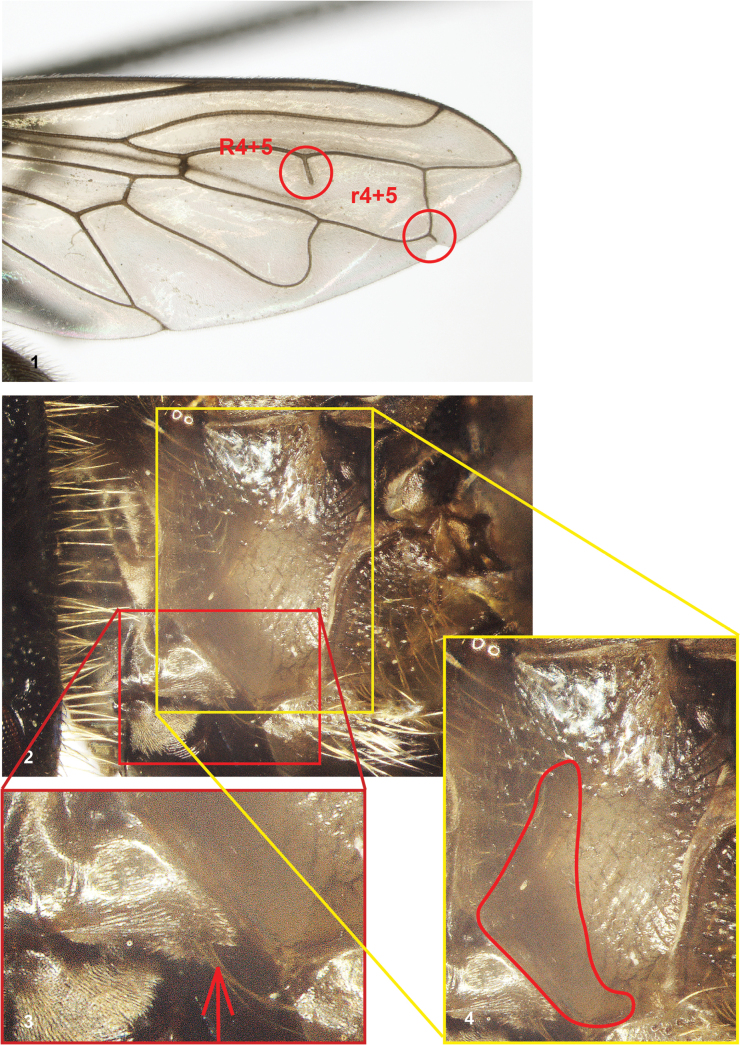
**1** wing of *Serichlamysvaricaudata* Reemer & Mengual, sp. nov. Note posterior appendix on vein R_4+5_ and near-rectangular (slightly acute) postero-apical corner pf cell r_4+5_**2–4** anterior pleura of *Serichlamysvaricaudata* Reemer & Mengual, sp. nov., lateral view **2** overview **3** proepisternum, note presence of setulae on the posterior part of this sclerite **4** anepisternum; note large bare medioventral part.

Body length: 5.5–12 mm; females generally larger than males. *Serichlamys* species are mostly dark coloured hoverflies with a more or less oval abdomen. The following combination of characters is unique to this genus: eye bare (setulose in *Laetodon* Reemer, 2013); vein R_4+5_ with posterior appendix extending into cell r_4+5_ (Fig. 1) (without such appendix in several other genera of Microdontinae); postero-apical corner of cell r_4+5_ more or less rectangular or somewhat acute (Fig. 1) (widely rounded in *Microdon* and *Peradon* Reemer, 2013); postpronotum setulose (bare in e.g., *Surimyia* Reemer, 2008 and certain species of *Peradon*); anepisternum with large bare part medially (Figs 2, 4) (entirely setulose in *Metadon* Reemer, 2013); proepisternum setulose (Figs 2, 3) (bare in *Archimicrodon* and many other genera); tergites 3 and 4 fused, not able to articulate independently (not fused in *Ceratophya* Wiedemann, 1830).

Species of *Serichlamys* look somewhat similar to those of the Old World genus *Archimicrodon*, with which they share most of the characters mentioned above. However, *Serichlamys* always differs in the presence of setulae on the proepisternum (bare in *Archimicrodon*). In general, the antennae of *Serichlamys* species are longer than those of *Archimicrodon* species (a character used in the key of Reemer and Ståhls 2013a), but there is considerable overlap, so this character is unreliable. In *Archimicrodon* the structure of the male genitalia is rather uniform across the species, with both phallus and surstylus generally of similar shape in all known species (Reemer and Ståhls 2013a: figs 22–26), whereas among species of *Serichlamys* there is much more variation (Figs 138–154). *Serichlamys* species also differ from *Archimicrodon* species in the presence of clearly defined patterns of dull and shiny areas on the tergites (in *Archimicrodon* the tergites are more uniformly shiny or dull). Interestingly, in a few *Serichlamys* species (e.g., *S.spathulata* Reemer, sp. nov.) the scutellar calcars are ‘spoon-shaped’, as in certain Afrotropical species of Archimicrodon (subgenus Hovamicrodon Keiser, 1971).

### ﻿Nomenclatural note

The genus name *Serichlamys* is feminine, as it is derived from the feminine Greek word *chlamys* meaning mantle, cloak (Brown 1956: 362; D. Yanega, pers. comm. 21 July 2023).

### ﻿Key to Neotropical species of *Serichlamys*

In many cases, external characters to distinguish between species are subtle and variable, often based on colouration or patterns of shiny vs. dull parts. Therefore, it is advised that the male genitalia are studied for more accurate determination. Identification of females remains uncertain in some cases, or even impossible in a few (*S.serpentiphallus* Reemer, sp. nov. versus *S.simpliciphallus* Reemer, sp. nov.), especially because for most species few female specimens are known, if any.

**Table:** 

1	Face dark brown to black, sometimes with metallic hues (Fig. 2) (in teneral or faded specimens the colour may be paler than usual)	**2**
–	Face yellowish or pale brown, without metallic hues (Fig. 3)	**9**
2	Tibiae with contrasting pattern of pale yellow basal 3/5, dark brown ring around cicatrix, and pale brown apex (Fig. 7). Male: phallus subapically with ventral large ‘flag-like’ projection (Figs 150–153)	***S.vexilliphallus* Reemer & Mengual, sp. nov.**
–	Tibiae more or less concolourous (yellowish brown, dark brown or blackish), although often darker around cicatrix (Figs 8, 9). Male: phallus without subapical ‘flag-like’ projection (Figs 138–144)	**3**
3	Tergite 3 either shiny with three dull maculae (a small median one and two larger lateral ones) [size varies and, sometimes, maculae are slightly interconnected or the median one is very small] (Figs 13, 15, 16), OR with one broad dull macula with three posterior ‘lobes’ or extensions (Fig. 14). Postpedicel pale brown with apical 1/3 dark brown (Fig. 10). Male genitalia as in Figs 142–144	**4**
–	Tergite 3 broadly dull with shiny margins (Fig. 17). Postpedicel uniformly coloured: entirely dark brown, blackish, or orange (Figs 11, 12)	**6**
4	Tarsi entirely dark brown to black (Fig. 18). Male genitalia: ventral lobe of surstylus without a median projection (Fig. 142). Female: scutellum yellow, strongly contrasting with dark scutum (Fig. 22); legs brownish yellow except tarsi dark brown to black (Fig. 20)	***S.melamitis* Reemer, sp. nov.**
–	Tarsi brown with apical tarsomeres yellowish brown (Fig. 19). Male genitalia: ventral lobe of surstylus with a median projection (Figs 143, 144). Female (note that female of *S.mellimitis* Reemer, sp. nov. is unknown, so characters may not apply): scutellum brown, of approximately same colour as scutum (Fig. 23); legs brown, tarsi not notably darker than femora and tibiae (Fig. 21)	**5**
5	Tergite 3 with three separate dull maculae (Figs 15, 16). Lateral margins of tergites 2–4 and posterior margin of tergite 4 brown, sometimes paler than median parts of tergites but never yellow (Fig. 82). Male genitalia as in Fig. 144	***S.mitis* (Curran)**
–	Tergite 3 with one large, wide dull macula with three posterior ‘lobes’ or extensions (Fig. 14). Lateral margins of tergites 2–4 and posterior margin of tergite 4 yellow, strongly contrasting with median parts of tergites (Figs 76, 77). Male genitalia as in Fig. 143. Female unknown	***S.mellimitis* Reemer, sp. nov.**
6	Wing cell dm with a large bare area posterobasally (Fig. 24). Male genitalia: surstylus with two basoventral setae placed close together (Fig. 138)	***S.boti* Reemer, sp. nov.**
–	Wing cell dm entirely microtrichose (Fig. 25). Male genitalia: surstylus with five or six basoventral setae grouped together on a small tubercle (Figs 139–141)	**7**
7	Scutum and scutellum with strong metallic green shine (Fig. 26); mesoscutum medially with sparse punctation, surface in between punctures smooth and shiny. Tergite 2 with pair of large, smooth, strongly metallic green shiny maculae (Fig. 31). Male genitalia as in Fig. 139	***S.chloraspis* Reemer, sp. nov.**
–	Scutum and scutellum with bronze or weak greenish shine (Fig. 27); mesoscutum medially with dense punctation, and surface in between punctures less shiny due to fine microsculpture. Tergite 2 with shiny parts not strongly metallic green (Figs 32, 33). Male genitalia as in Figs 140, 141	**8**
8	Tergite 2 with lateral dull maculae small, narrower than the shiny areas in between (Fig. 32) (this character may be difficult to assess in wet or greasy specimens and should be viewed from multiple angles and lighting combinations). Hind metatarsus dorsally orange-brown (Fig. 28). Female: postpedicel orange (Fig. 34). Male: genitalia as in Fig. 140	***S.pallitarsis* Reemer & Mengual, sp. nov.**
–	Tergite 2 with lateral dull parts large, wider than shiny areas in between (Fig. 33). Hind metatarsus dorsally black (Fig. 29). Female: postpedicel blackish, except base pale brown (Fig. 35). Male genitalia as in Fig. 141	***S.varicaudata* Reemer & Mengual, sp. nov.**
9	(2) Abdomen with greatest width at approximately middle of tergite 2 (Fig. 36)	***S.trigonoides* Reemer, sp. nov.**
–	Abdomen with greatest width at transition of tergites 2 and 3, or beyond (Fig. 37)	**10**
10	Femora black on basal 2/3, legs otherwise yellowish brown (Fig. 38). Alula almost entirely microtrichose (only narrow strip at base bare). Male genitalia as in Fig. 154	***S.xanthocnemia* Reemer, sp. nov.**
–	Legs entirely brown, with femora usually somewhat darker than tibiae (Fig. 39). Alula with large basomedian part bare. Male genitalia as in Figs 146–149	**11**
11	Tergite 2 with a wide fascia of grey microtrichia medially (viewed from behind as in Figs 46, 47); sometimes with at most a pair of small round bare areas at sides (Fig. 47), defining a median microtrichose area wider than the submedian shiny parts. Postpedicel black, except sometimes basal 1/4 brown (Fig. 41). Male genitalia as in Fig. 147	***S.spathulata* Reemer, sp. nov. ^1^**
–	Tergite 2 with two or three separate patches of grey microtrichia (viewed from behind as in Figs 44, 45, 48, 49), with submedian shiny areas wider than the median grey-microtrichose patch. Postpedicel of variable colouration (Figs 40, 42, 43). Male genitalia as in Figs 146, 148, 149	**12**
12	Male genitalia as in Fig. 146. Scutellar calcars narrow with acute apex (Fig. 50) (note that only two males and one likely female of this species are known, so variability of the shape of the scutellar calcars is insufficiently known). Postpedicel orange with apical 1/4 dark (Fig. 40)	***S.mus* (Curran)**
–	Male genitalia as in Figs 147–149. Scutellar calcars often (but not always) broad and flattened, spoon-shaped (Figs 53–57). Postpedicel of variable colouration (Figs 42, 43)	**12**
13	Male genitalia: phallus strongly curved, more or less S-shaped, with apex unfurcate (Fig. 148). Male: postpedicel usually dark, except sometimes basal 1/4 paler (Fig. 42). Females undistinguishable from next species	***S.serpentiphallus* Reemer, sp. nov.**
–	Male genitalia: phallus more or less straight (Fig. 149). Male: postpedicel orange with apical 1/4 dark (Fig. 43). Females undistinguishable from previous species	***S.simpliciphallus* Reemer, sp. nov.**

**Figures 5–12.**
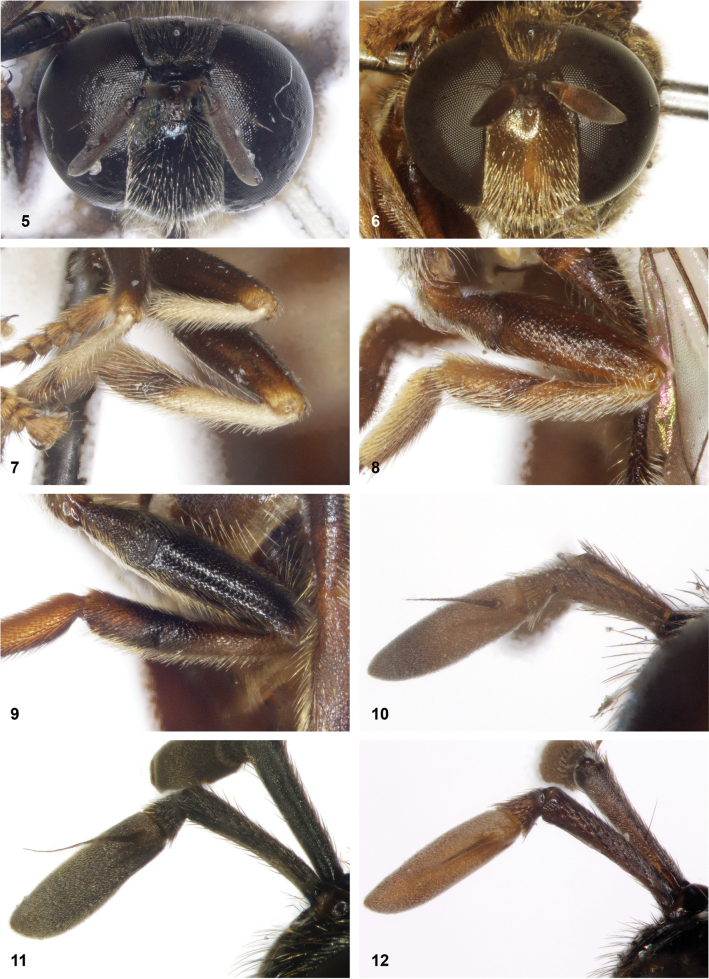
**5, 6** colour of face of *Serichlamys* species **5** black (*S.chloraspis* Reemer, sp. nov. male, holotype) **6** yellowish brown (*S.serpentiphallus* Reemer, sp. nov. male, holotype) **7–9** hind tibiae of *Serichlamys* species **7** with contrasting colour pattern (*S.vexilliphallus* Reemer & Mengual, sp. nov. male, holotype) **8** uniformly yellowish brown (*S.mitis* (Curran, 1940) male, specimen MR1576) **9** dark (*S.pallitarsis* Reemer & Mengual, sp. nov. male, holotype) **10**–**12** antenna of *Serichlamys* species **10** postpedicel pale brown with apical 1/3 dark (*S.mitis* (Curran, 1940) male, specimen MR1576) **11** postpedicel entirely dark brown (*S.varicaudata* Reemer & Mengual, sp. nov. male, holotype) **12** postpedicel entirely orange (*S.pallitarsis* Reemer & Mengual, sp. nov. female, paratype).

**Figures 13–17.**
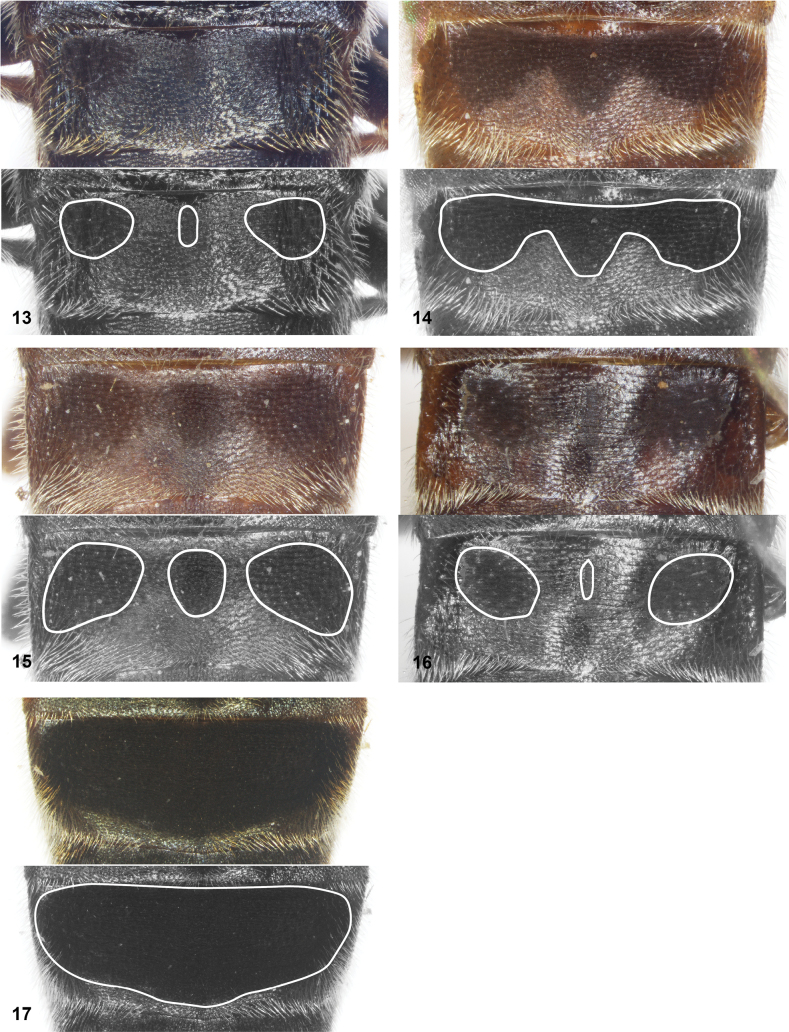
Tergite 3 of *Serichlamys* species. White lines in lower half of figures indicate position of dull maculae **13***S.melamitis* Reemer, sp. nov. male, holotype **14***S.mellimitis* Reemer, sp. nov. male, holotype **15***S.mitis* (Curran, 1940) male, specimen MR1576 **16***S.mitis* (Curran, 1940) female, specimen MR1581 **17***S.varicaudata* Reemer & Mengual, sp. nov. male, holotype.

**Figures 18–23.**
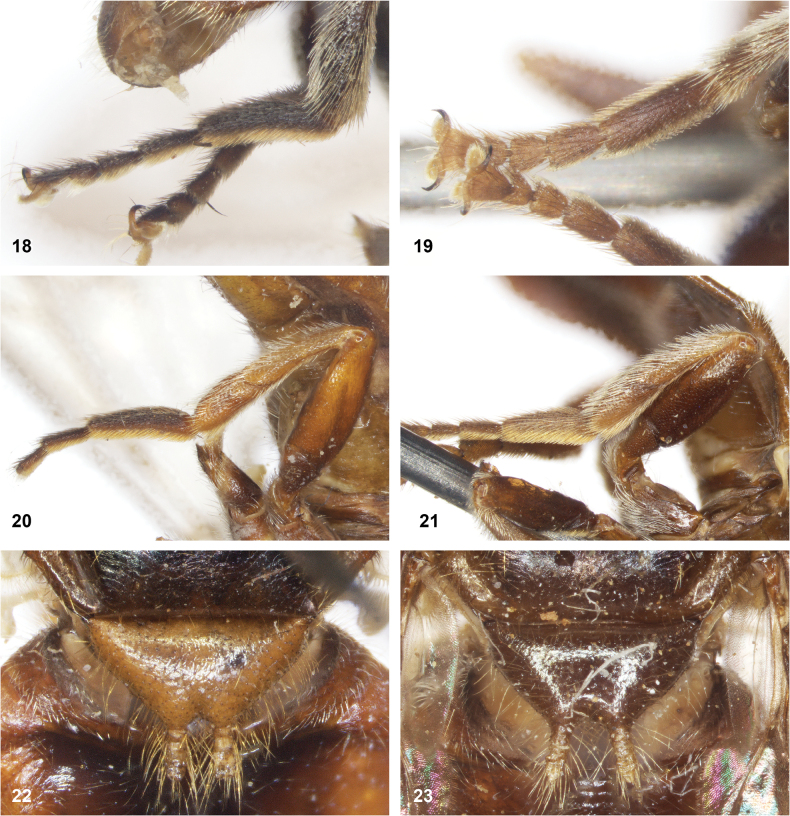
**18, 19** hind tarsus of *Serichlamys* males **18***S.melamitis* Reemer & Mengual, sp. nov. holotype **19***S.mitis* (Curran, 1940) specimen MR1576 **20, 21** hind leg of *Serichlamys* females **20***S.melamitis* Reemer & Mengual, sp. nov. paratype **21***S.mitis* (Curran, 1940) specimen MR1581 **22, 23** scutellum of *Serichlamys* females **22***S.melamitis* Reemer & Mengual, sp. nov. paratype **23***S.mitis* (Curran, 1940) specimen MR1581.

**Figures 24–29.**
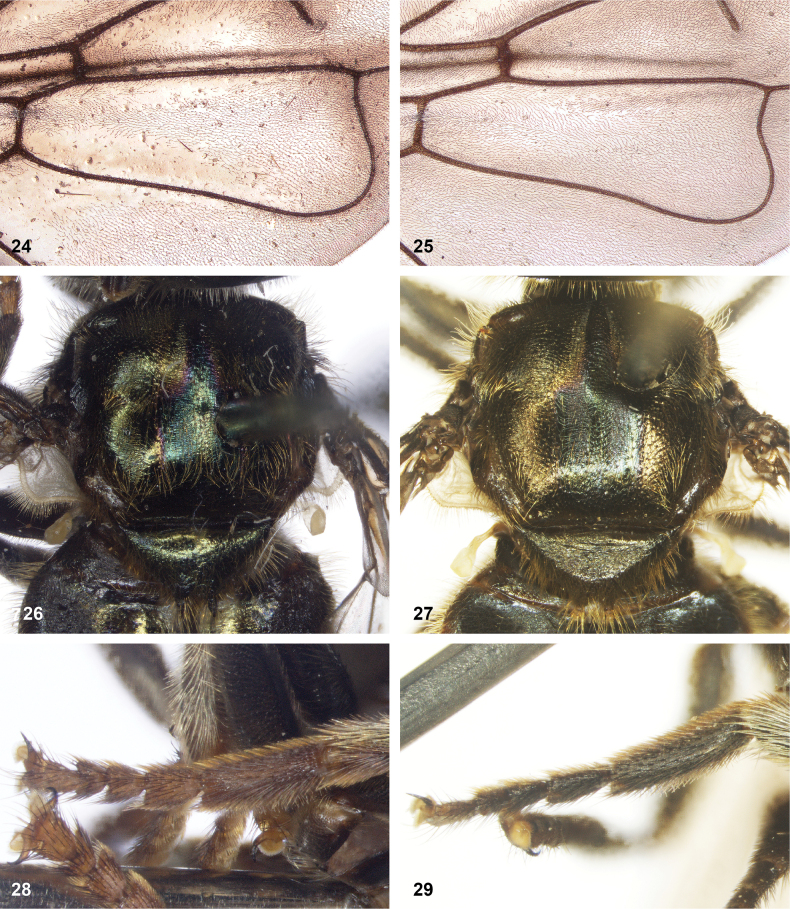
**24, 25.** Microtrichiae on wing cell dm in *Serichlamys* species **24***S.boti* Reemer, sp. nov. male, holotype **25***S.varicaudata* Reemer & Mengual, sp. nov. male, holotype **26, 27** scutum and scutellum of *Serichlamys* males **26***S.chloraspis* Reemer, sp. nov. holotype **27***S.varicaudata* Reemer & Mengual, sp. nov. holotype **28, 29** hind tarsus of *Serichlamys* males **28***S.pallitarsis* Reemer & Mengual, sp. nov. holotype **29***S.varicaudata* Reemer & Mengual, sp. nov. holotype.

**Figures 30–35.**
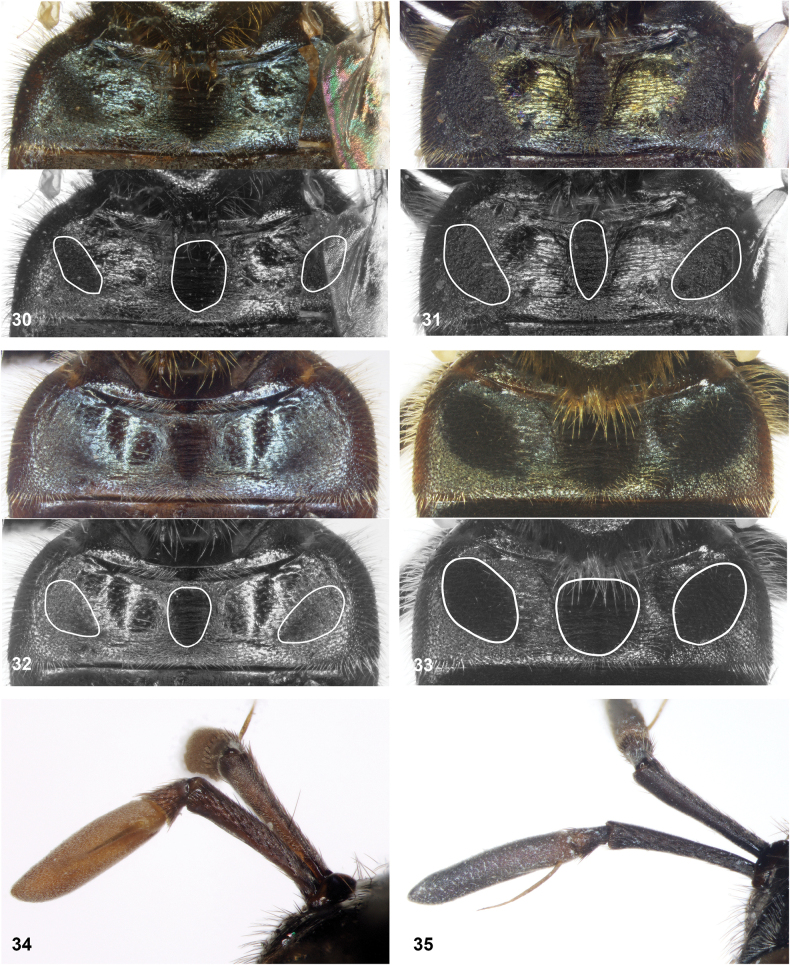
**30–33** tergite 2 of *Serichlamys* males. White lines in lower half of figures indicate position of dull maculae **30***S.boti* Reemer, sp. nov. holotype **31***S.chloraspis* Reemer, sp. nov. holotype **32***S.pallitarsis* Reemer & Mengual, sp. nov. holotype **33***S.varicaudata* Reemer & Mengual, sp. nov. holotype **34, 35** antenna of *Serichlamys* females **34***S.pallitarsis* Reemer & Mengual, sp. nov. paratype **35***S.varicaudata* Reemer & Mengual, sp. nov. paratype.

**Figures 36–43.**
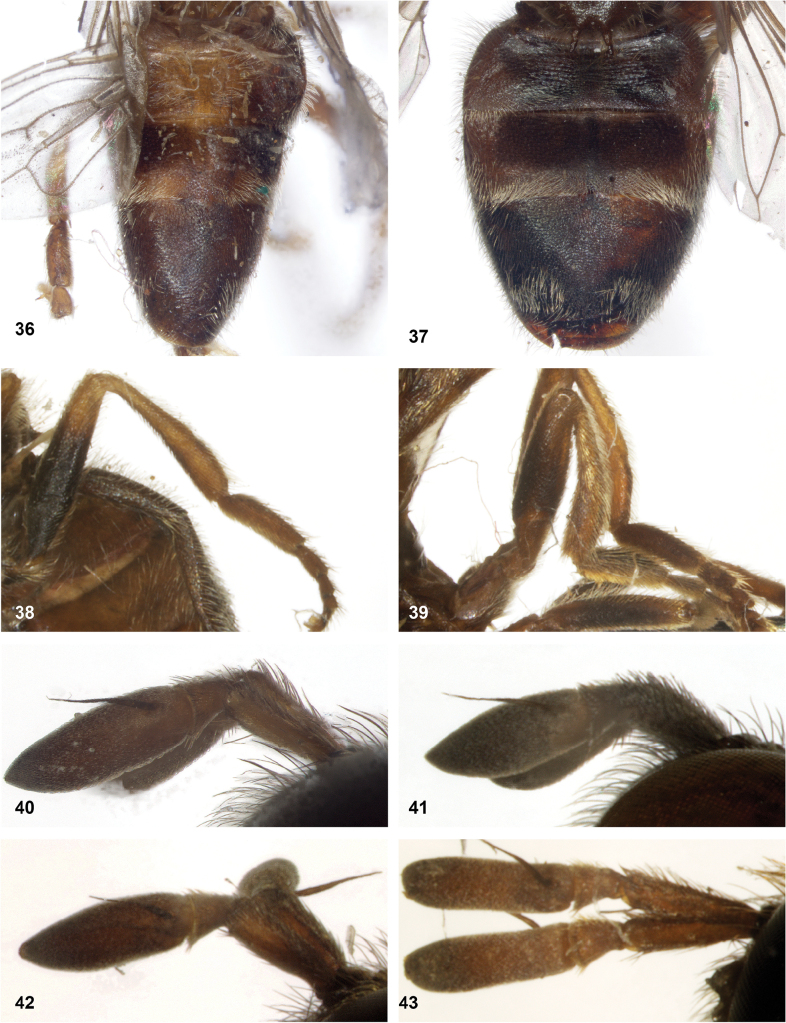
**36, 37** abdomen of *Serichlamys* males **36***S.trigonoides* Reemer, sp. nov. holotype **37***S.mus* (Curran, 1936) specimen USNMENT01866933 (= MR1587) **38, 39** hind leg of *Serichlamys* males **38***S.xanthocnemia* Reemer, sp. nov. holotype **39***S.simpliciphallus* Reemer, sp. nov. holotype **40–43** antenna of *Serichlamys* males (note that shape of postpedicel may vary within species, with apex acute or rounded) **40***S.mus* (Curran, 1936) specimen MR1587 **41***S.spathulata* Reemer, sp. nov. holotype **42***S.serpentiphallus* Reemer, sp. nov. holotype **43***S.simpliciphallus* Reemer, sp. nov. holotype.

**Figures 44–49.**
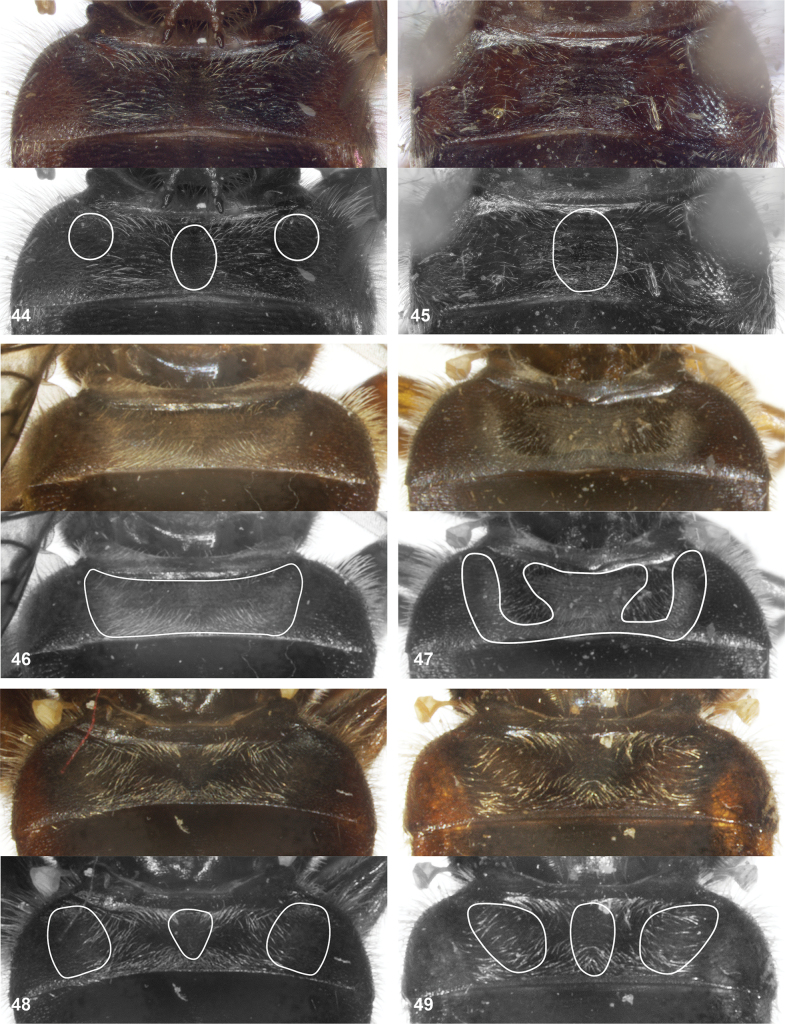
Tergite 2 of *Serichlamys*, posterodorsal view. White lines in lower half of figures indicate position of patches of grey microtrichia (vary with viewing angle and lighting to get good view on shiny and dull parts) **44***S.mus* (Curran, 1936) male, specimen MR1587 **45***S.mus* (Curran, 1936) female, specimen MR1606 **46***S.spathulata* Reemer, sp. nov. male, holotype **47***S.spathulata* Reemer, sp. nov. female, paratype **48***S.serpentiphallus* Reemer, sp. nov. male, holotype **49***S.simpliciphallus* Reemer, sp. nov. male, holotype.

**Figures 50–57.**
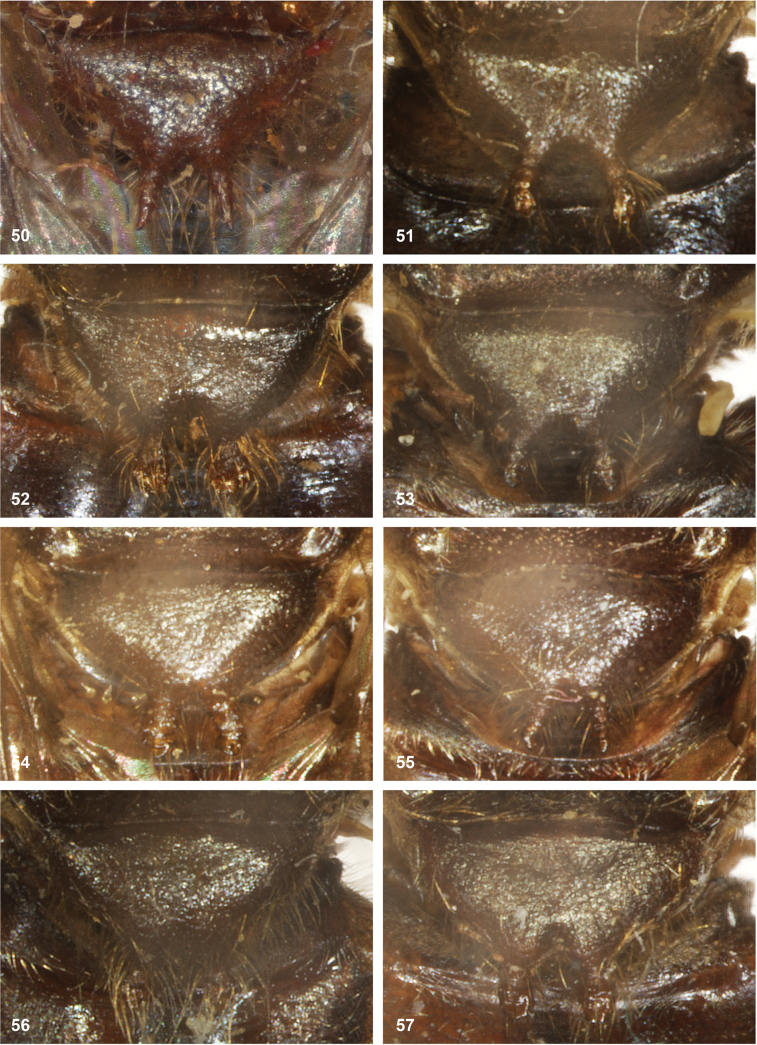
Scutellar calcars of *Serichlamys***50***S.mus* (Curran, 1936) male, holotype **51***S.spathulata* Reemer, sp. nov. holotype, male **52***S.spathulata* Reemer, sp. nov. female, paratype **53***S.serpentiphallus* Reemer, sp. nov. male, holotype **54***S.serpentiphallus* Reemer, sp. nov. male, paratype **55***S.serpentiphallus* Reemer, sp. nov. male, paratype **56***S.simpliciphallus* Reemer, sp. nov. male, holotype **57***S.simpliciphallus* Reemer, sp. nov. male, paratype 1957 MZUSP.

**Figures 58–63.**
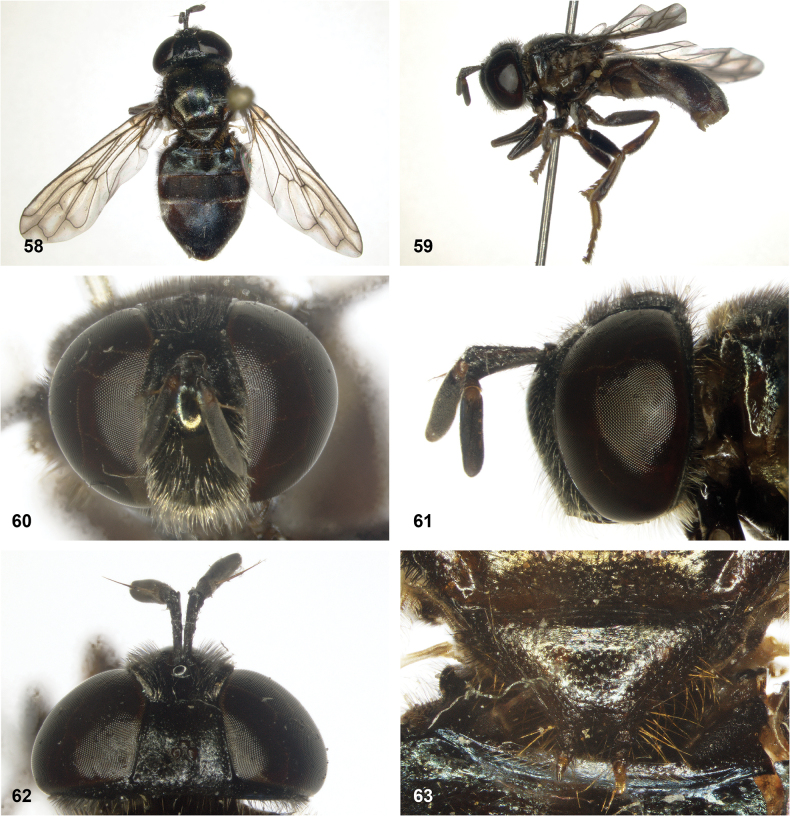
*Serichlamysboti* Reemer, sp. nov. male, holotype **58** habitus, dorsal **59** habitus, lateral **60** head, frontal **61** head, lateral **62** head, dorsal **63** scutellum, dorsal.

### ﻿Species accounts

#### ﻿ Serichlamys boti

Taxon classificationAnimaliaDipteraSyrphidae

Reemer sp. nov.

9280DAEB-DBAA-5ECA-B5F8-CCCEDBAC3678

https://zoobank.org/892B3273-6FAD-4FF7-99BE-DAE9A29360AB

Figs 24 , 30 , 58–63 , 138

##### Type material.

***Holotype*.** Colombia • 1 ♂, holotype of *Serichlamysboti* sp. nov.; Risaralda, Tatamá National Park; 1481 m; 5.239°N, 76.090°W; 4 Mar. 2015; S. Bot leg.; CEUA.

Label 1: “CO Risaralda // Tatamá NP, 1481 m // lat5.239 lng -76.090 // 4 III 2015 leg. S. Bot”; label 2: “Serichlamys sp. nov. // det. M. Reemer // specimen code MR1485”. ***Paratypes*.** Colombia • 1 ♂; same locality and date as holotype; specimen code MR0596; CEUA. Ecuador • 4 ♂; Pinchincha, Nambillo Valley near Mindo; 1450 m; 26 Jun. 1987; M. Cooper leg.; NHMUK (barcodes on labels: NHMUK013624730, NHMUK013624731, NHMUK013624732, NHMUK013624733) • 3 ♀; Pinchincha, Nambillo Valley near Mindo; 1450 m; one from 27 Jun. 1987 (NHMUK013624724), one from 28 Jun. 1987 (NHMUK013624726), one from 30 Jun. 1987 (NHMUK013624725); M. Cooper leg.; NHMUK.

##### Description

**(based on holotype). Adult male** Body size: 9.5 mm.

***Head*.** Face occupying around 2/5 of head width in frontal view; shiny black; white setulose laterally, black setulose sublaterally, bare at median part as wide as ~ 1/4 of width of face. Gena very narrow; black; white setulose. Oral margin laterally not produced. Frons black; medially bare, laterally black setulose. Vertex black; black setulose. Occiput black; dorsal 1/2 black setulose, ventral 1/2 white setulose. Eye bare. Antenna black, except pedicel and basal 1/6 of postpedicel brown; antennal ratio ~ 7:1:7.5.

***Thorax*.** Scutum shiny black, with bronze sheen on lateral 1/3 and faint dark blue sheen on median 1/3; black setulose, except for narrow fascia of golden yellow setulae along anterior margin. Postpronotum of same colour as scutum; black and dark yellow setulose. Postalar callus dark brown; black setulose. Scutellum trapezoid, with two calcars as long as ~ 1/3 of scutellar length; of same colour as scutum except calcars largely pale brown; black setulose anteriorly and medially, yellow setulose laterally and posteriorly. Pleura shiny blackish brown, except anepimeron and katepimeron pale brown. Anepisternum with shallow sulcus; dark yellow setulose anteriorly, black setulose posteriorly, widely bare medially and ventrally. Anepimeron black setulose anteriorly, dark yellow setulose posteriorly. Katepisternum with patch of pale yellow setulae dorsally and small patch of yellow setulae ventrally. Katatergite long microtrichose, anatergite short microtrichose. Calypter greyish yellow and halter pale yellow.

Wing: hyaline; microtrichose, except bare on basal 1/3 of cell r_1_, a small basal patch of r_2+3_, most of cell br (only microtrichose along vena spuria), most of cell bm except for narrow median strip of microtrichiae, large part of cell dm (Fig. 24), anterobasal 1/2 of cell cup, and basomedian 3/4 of alula.

Legs: femora black, only apex brown; black setulose. Tibiae brown at both ends, blackish in between; largely black setulose, with some pale setulae ventrally. Tarsi orange-brown; black setulose dorsally, golden yellow setulose ventrally. Coxae and trochanters blackish; fore and mid coxae black setulose anteriorly, pale setulose posteriorly; hind coxa silvery white setulose.

***Abdomen*.** Tergites black to blackish brown, slightly paler along lateral margins. Tergite 1 black setulose. Tergite 2 shiny, except for oval median dull macula and pair of smaller, oblique lateral maculae; black setulose, except yellow setulose in anterolateral corners, and white setulose along posterior margin. Tergite 3 dull, except for narrow shiny strips along lateral and posterior margins; black setulose, except narrowly white setulose along posterior margin. Tergite 4 shiny; black setulose, except white setulose in anterolateral corners and on pair of larger posterior patches. Sternites blackish; sternites 1–3 white, setulose, sternite 4 black setulose. Genitalia as in Fig. 138.

**Female.** Unknown.

##### Diagnosis.

Body length: male 9.5–10 mm (*n* = 2). Superficially, this species looks most similar to *S.chloraspis* Reemer, sp. nov., *S.pallitarsis* Reemer & Mengual, sp. nov., and *S.varicaudata* Reemer & Mengual, sp. nov., which are of similar size and colouration. These species also share the following combination of characters: face black, tibiae entirely brown, tergite 3 broadly dull with narrow shiny margins (Fig. 58). *Serichlamysboti* Reemer, sp. nov. differs from the three other species, as well as from all other known species of *Serichlamys*, in the partly bare wing cell dm (Fig. 24). It also differs from the three species mentioned above in the presence of only two basoventral setae on the surstylus (Fig. 138) (instead of 4 or 5 in the other three species, Figs 139–141). *Serichlamysboti* Reemer, sp. nov. is similar to *S.pallitarsis* Reemer & Mengual, sp. nov. in the pattern of dull maculae on tergite 2: dull maculae narrower than shiny areas separating them (wider than shiny areas in *S.chloraspis* Reemer, sp. nov. and *S.varicaudata* Reemer & Mengual, sp. nov.) (compare Figs 31, 33). *Serichlamysboti* Reemer, sp. nov. and *pallitarsis* Reemer & Mengual, sp. nov. also share orange tarsi (dark in *S.chloraspis* Reemer, sp. nov. and *S.varicaudata* Reemer & Mengual, sp. nov.). However, the partly bare wing cell dm (entirely microtrichose in *S.pallitarsis*) and the two basoventral setae on the surstylus (4 or 5 in *S.pallitarsis* Reemer & Mengual, sp. nov.) clearly separate this species.

The holotype and the paratype differ in the colour of the setulae on the scutum: black except for narrow fascia of golden yellow setulae along anterior margin in the holotype, entirely golden yellow in the paratype.

##### Etymology.

The name of this species is a patronym in honour of Sander Bot, who collected the holotype and one of the paratypes.

##### Distribution.

This species is known from two localities on the western slope of the Andes: one in Colombia (elevation 1481 m), and one locality in Ecuador (elevation 1450 m).

#### ﻿ Serichlamys chloraspis

Taxon classificationAnimaliaDipteraSyrphidae

Reemer sp. nov.

3C87FDB1-2F7B-5B25-BAC7-DA126686EA09

https://zoobank.org/5D706E57-9997-406A-A492-2A3E921BA4F3

Figs 5 , 26 , 31 , 64–68 , 139

Microdon CR-99 Thompson, in litt. [see below under Molecular data]
Microdon MRC20 Thompson, in litt. [see below under Molecular data]

##### Type material.

***Holotype*.** Costa Rica • 1 ♂, holotype of *Serichlamyschloraspis* sp. nov.; Los Cruces; 8.78578°N, 82.95995°W; 30 Jul. 2010; J.H. Skevington leg.; CNC. Label 1: “COSTA RICA: Los Cruces // 30.vii.2010; 1191 m. // 8.78578 °N, 82.95995 °W // J.H. Skevington”; label 2: “J. Skevington // Specimen # // 22180”; label 3 (green): “Barcode of Life // DNA voucher specimen // Sample ID: JSS22180 // BOLD Proc. ID: CNCDB3735-11”; label 4: “*Serichlamys* sp. // Det. M. Reemer 2016 // Voucher code MR 1175” [GenBank accession no. PQ628996]. ***Paratypes*.** Costa Rica • 1 ♂; Puntarenas, Osa Peninsula, 2.5 km S Rincón; 8°42'1"N, 83°30'50"W; 50 m asl; 10–11 Aug. 2001; S.A. Marshall leg.; coll. DEBU; DNA voucher code MZH:S264; specimen code M. Reemer MR186; GenBank accession no. EU431495 • 1 ♂; Puntarenas, Peninsula de Osa; 8°40'50"N, 83°31'32"W; June 1998; B. Brown leg.; coll. UCRC [specimen code UCRC ENT 69104].

##### Additional specimens.

Costa Rica • 3 ♂; Higuito, San Mateo; Pablo Schild leg.; USNM [USNM specimen barcodes: USNMENT01866950, USNMENT01866950, USNMENT01866952] • 1 ♀; Puntarenas, Area de conservacion Osa, Golfito, Sector La Leona, Cerro Puma, 8.455°N, 83.495°W, 200 m asl, 17 Sep. 2003, M. Moraga leg.; specimen code MNCR-A3780889 (=INB0003780889); BOLD Process ID ASIND083-12; GenBank accession no. PQ629016; coll. MNCR.

##### Description

**(based on holotype). Adult male** Body size: 10 mm.

***Head*.** Face occupying slightly less than 1/3 of head width in frontal view; shiny black; white setulose, except narrow bare patch below antennae. Gena very narrow; black; white setulose. Oral margin laterally not produced. Frons black; medially bare, laterally black setulose. Vertex black; black setulose except for small patch of golden yellow setulae anteriorly and a few scattered golden yellow setulae posteriorly. Occiput black; dorsal 1/3 golden yellow setulose, otherwise white setulose. Eye bare. Antenna blackish brown; antennal ratio ~ 4:1:5.

***Thorax*.** Scutum shiny black with clear green sheen; golden yellow setulose, except for small median patch of black setulae on anterior 1/3 and some scattered black setulae laterally posteriad of transverse suture. Postpronotum of same colour as scutum; golden yellow setulose. Postalar callus brown; yellow setulose with a few black setulae ventrolaterally. Scutellum trapezoid, with two calcars as long as ~ 1/4 of scutellar length; of same colour as scutum except calcars dull black; golden yellow setulose with a few black setulae posteriorly. Pleura shiny blackish brown. Anepisternum with shallow sulcus; golden yellow setulose anterodorsally, black setulose posterodorsally, widely bare medially and ventrally. Anepimeron golden yellow setulose with a few black setulae dorsally. Katepisternum with patch of pale yellow setulae dorsally and small patch of whitish setulae ventrally. Katatergite long microtrichose, anatergite short microtrichose. Calypter and halter pale yellow.

Wing: hyaline; microtrichose, except bare on basal 1/8 of cell r_1_ (along vein RS), most of cell br (only microtrichose along vena spuria), posterobasal 1/3 of cell bm, anterobasal 1/3 of cell cup, and basomedian 1/2 of alula.

Legs: femora and tibiae black, with tibiae a little brownish at both ends; tarsi brown, with apical tarsomeres paler than basal four tarsomeres. Femora black setulose, with ventrobasal patches of pale setulae. Tibiae black setulose anteriorly, white setulose posteriorly. Coxae and trochanters blackish brown; white setulose.

***Abdomen*.** Tergites black. Tergite 1 yellow setulose, except black setulose near lateral margin. Tergite 2 golden yellow setulose, except for two large, bare, strongly metallic green shiny maculae, and black setulose along posterior half of lateral margin. Tergite 3 on most of surface with a mixture of short erect golden yellow and black setula; golden yellow setulae along posterior and lateral margins longer and more appressed, resulting in a fascia along these margins. Tergite 4 mostly short appressed black setulose, but longer pale yellow to whitish setulose along lateral margins and on pair of large posterior patches. Sternites blackish brown; sternite 1 bare; sternites 2 and 3 yellowish white setulose; sternite 4 black setulose. Genitalia as in Fig. 139.

**Female.** Unknown. The female listed under Additional material is included based on its COI barcode, but the specimen itself was not studied, as it was not possible for us to arrange a loan within the time frame of this study.

##### Diagnosis.

**Figures 64–68.**
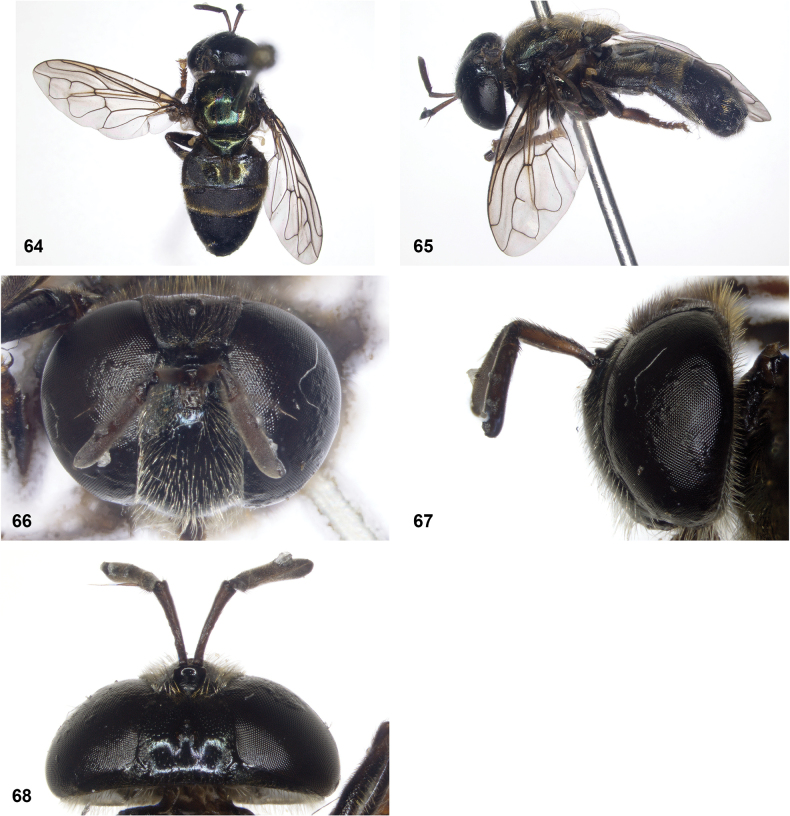
*Serichlamyschloraspis* Reemer, sp. nov. male, holotype **64** habitus, dorsal **65** habitus, lateral **66** head, frontal **67** head, lateral **68** head, dorsal.

Body length: male 8–10 mm (*n* = 6). Superficially, this species looks most similar to *S.boti* Reemer, sp. nov., *S.pallitarsis* Reemer & Mengual, sp. nov. and *S.varicaudata* Reemer & Mengual, sp. nov., which are of similar size and colouration. These species also share the following combination of characters: face black, tibiae entirely brown, tergite 3 broadly dull with narrow shiny margins (Fig. 64). However, this is the only known species of *Serichlamys* with a strong metallic green shine on scutum and scutellum (Figs 31, 64). A weak metallic, sometimes green, shine also occurs in other species, but not as strong. In addition, there is a pair of large, smooth, metallic green maculae on tergite 2 (Fig. 31) (these maculae are not green in the three species mentioned above).

##### Etymology.

The specific epithet is composed of the Greek words *chloros* (green) and *aspis* (shield). This name refers to the metallic green scutum and scutellum of this species.

##### Molecular data.

The holotype male was successfully sequenced and its DNA barcode clusters together (BS = 100) with the COI sequence of one of the paratypes (MZH S264), which had previously been identified as ‘*Microdon* CR99-10’ in GenBank, and a public sequence of another *Serichlamys* specimen from Costa Rica, previously identified as *Microdon* MRC-20. Both codes or name-holders are known to be used by F.C. Thompson and likely refer to the same species, *S.chloraspis* Reemer, sp. nov.

##### Distribution.

This species is only known from Costa Rica, where it was found in the centre and southeast of the country at elevations between 50 and 1200 meters.

#### ﻿ Serichlamys melamitis

Taxon classificationAnimaliaDipteraSyrphidae

Reemer sp. nov.

0B298859-3316-5805-8354-41586D335C25

https://zoobank.org/F703863F-6AD6-49D1-8EC2-5374CDD20A0C

Figs 13 , 18 , 69–75 , 142

##### Type material.

***Holotype*.** Brazil • 1 ♂, holotype of *Serichlamysmelamitis* sp. nov.; Salesópolis, Estação Biológica de Boraceia; 850 m asl; 5 Aug. 1964; Rabello leg.; MZUSP. Label 1: “EST. BIOL. BORACEIA // Salesópolis, S.P. 850 m. // Rebello col. 5.VIII.1964”; label 2: “Serichlamys sp. // Det. M. Reemer 2024 // Specimen code MR1594”. ***Paratypes*.** Brazil • 3 ♂ 1 ♀ of same locality and date as holotype, 2 ♂ (M. Reemer specimen codes MR1591 and MR1593) and 1 ♀ in MZUSP, 1 ♂ in RMNH (M. Reemer specimen code MR1592).

##### Description

**(based on holotype). Adult male** Body size: 8 mm.

***Head*.** Face occupying ~ 1/3 of head width in frontal view; shiny black; white setulose, except black setulose on dorsal 1/4. Gena very narrow; black; white setulose. Oral margin laterally not produced. Frons black; medially bare, laterally black setulose. Vertex black; black setulose on anterior half, except for a few golden yellow setulae anteriorly, golden yellow setulose on posterior half. Occiput black; dorsal 1/2 golden yellow setulose, ventral 1/2 white setulose. Eye bare. Antenna orange-brown, except apical 1/3 of postpedicel black; antennal ratio ~ 2.5:1:4.5.

***Thorax*.** Scutum shiny black with faint metallic sheen; golden yellow setulose. Postpronotum of same colour as scutum; golden yellow setulose. Postalar callus pale brown; yellow setulose. Scutellum trapezoid, of same colour as scutum, with two pale yellow calcars as long as ~ 1/2 of scutellar length. Pleura shiny brown, except meron and ventral parts of katepisternum blackish. Anepisternum with shallow sulcus; golden yellow setulose anterodorsally and posterodorsally, widely bare medially and ventrally. Anepimeron golden yellow setulose. Katepisternum with patch of white setulae dorsally and a few white setulae ventrally. Katatergite long microtrichose, anatergite short microtrichose. Calypter and halter pale yellow.

Wing: hyaline; microtrichose, except bare on basal 1/4 of cell r_1_ (along vein RS), most of cell br (only microtrichose along vena spuria), posterobasal 2/5 of cell bm, anterobasal 2/5 of cell cup, and basomedian 1/2 of alula.

Legs: femora blackish brown, with apices narrowly yellowish; yellowish setulose except black setulose anterodorsally. Tibiae dark brown with vaguely demarcated black rings; yellowish setulose. Fore tarsus yellow setulose, mid tarsus yellow setulose except apical tarsomeres dorsally black setulose, hind tarsus black setulose dorsally and yellow setulose ventrally. Coxae and trochanters blackish brown; yellowish white setulose.

***Abdomen*.** Tergites black, except lateral margins and posterior margin of tergite 4 brown. Tergite 1 yellowish white setulose. Tergite 2 golden yellow setulose; strongly shiny medially, semi-shiny on lateral 1/4 due to microsculpture. Tergite 3 semi-shiny on most of surface, with small round maculae laterally and a smaller dull macula medially; golden yellow setulose except black setulose medially. Tergite 4 semi-shiny; golden yellow setulose laterally and posteriorly, black setulose anteriorly and medially. Sternites brown; white setulose. Genitalia as in Fig. 142.

**Female.** As male, except for following differences. Body length 11 mm. Scutellum yellow, strongly contrasting with scutum (Fig. 74). Tergites with lateral margins widely yellowish. Tergite 5 largely yellowish (Fig. 75). Legs pale brown, except tarsi dark brown.

**Figures 69–75.**
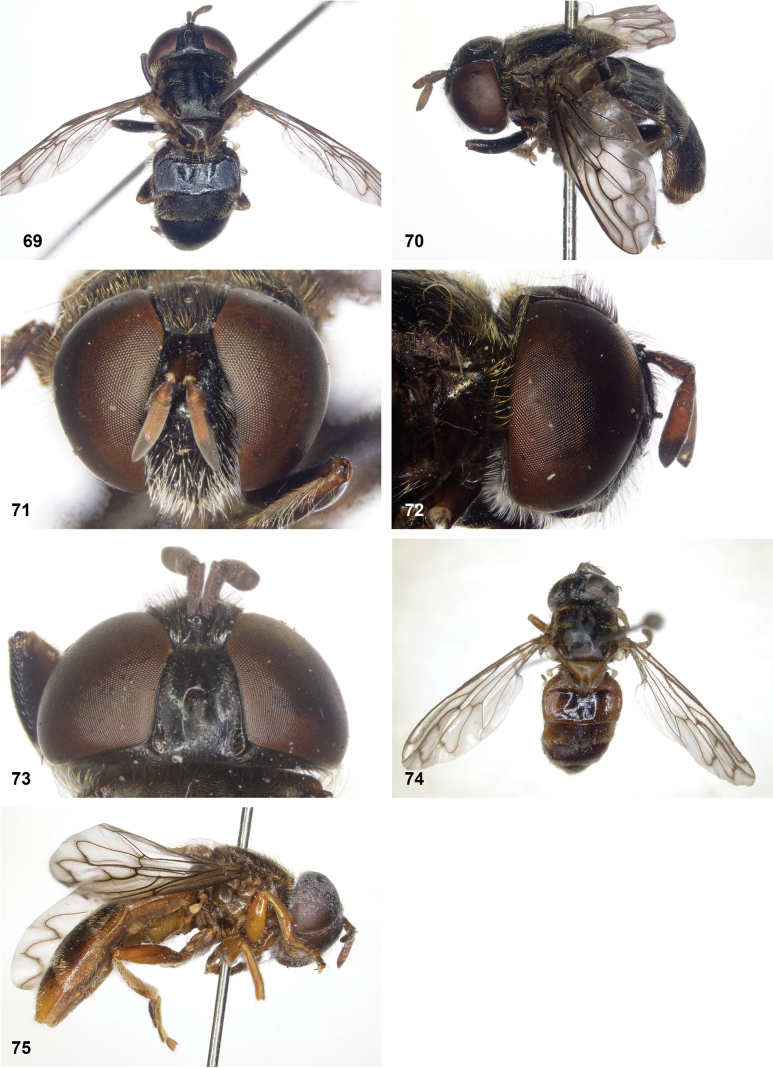
**69–73.***Serichlamysmelamitis* Reemer, sp. nov. male, holotype **69** habitus, dorsal **70** habitus, lateral **71** head, frontal **72** head, lateral **73** head, dorsal **74, 75***Serichlamysmelamitis* Reemer, sp. nov. female, paratype **74** habitus, dorsal **75** habitus, lateral.

##### Diagnosis.

Body length: male 8–8.5 mm (*n* = 4), female 11 mm (*n* = 1). Together with *S.mellimitis* Reemer, sp. nov. and *S.mitis*, this species combines a black face with antennae in which the postpedicel has a dark tip (Figs 71, 72). This species differs from the other two by its entirely dark tarsi (Fig. 18), and the absence of a median projection on the ventral lobe of the surstylus (Fig. 142). In the female, the scutellum is yellow, contrasting strongly with the dark scutum (Fig. 74).

##### Etymology.

The species name refers to the species *Serichlamysmitis* (Curran), to which this new species is very similar. The prefix *mela* (from the Greek *melas*, black) was chosen because this species is more blackish in colouration than *S.mitis*.

##### Distribution.

This species is only known from Salésopolis in the Brazilian State of São Paulo.

#### ﻿ Serichlamys mellimitis

Taxon classificationAnimaliaDipteraSyrphidae

Reemer sp. nov.

D42B8701-F5A6-5A8A-99DB-DFD29A8E3FCD

https://zoobank.org/831A1283-040F-46F9-917F-9479E98A178C

Figs 14 , 76–81 , 143

##### Type material.

***Holotype*.** Brazil • 1 ♂, holotype of *Serichlamysmellimitis* sp. nov.; Minas Gerais, Belo Horizonte, Pampulha, Univ. Fed. Minas Gerais; 19°52'S, 43°58'W; 23 Oct. – 2 Nov. 1996; yellow pan trap; D. Yanega leg.; UCRC. Label 1: “BRASIL: Minas Gerais // Belo Horizonte, Pampulha // Univ. Fed. Minas Gerais // 19°52’ S 43°58’ W, YPT // 23.x-2.xi.1996 D. Yanega”; label 2: “Univ. Calif. Riverside // Ent. Res. Museum // UCRC ENT 71927”. ***Paratypes*.** Brazil • 3 ♂ of same locality and date as holotype; 2 in coll. UCRC [UCRC ENT 71926 and UCRC ENT 71928], 1 in coll. RMNH [UCRC ENT 71929].

##### Description

**(based on holotype). Adult male** Body size: 6.5 mm.

***Head*.** Face occupying ~ 1/3 of head width in frontal view; shiny black; white setulose. Gena very narrow; black; white setulose. Oral margin laterally not produced. Frons black; medially bare, laterally black, and white setulose. Vertex black; golden yellow setulose anteriorly and posteriorly, black setulose in between. Occiput black; dorsal 1/2 golden yellow setulose, ventral 1/2 white setulose. Eye bare. Antenna orange-brown, except apical 1/3 of postpedicel darker; antennal ratio ~ 4:1:5.

***Thorax*.** Scutum shiny black with bronze sheen, margins brown; golden yellow setulose. Postpronotum and postalar callus brown; golden yellow setulose. Scutellum trapezoid, brown, with two pale yellow calcars as long as ~ 1/2 of scutellar length; golden yellow setulose. Pleura shiny brown. Anepisternum with shallow sulcus; golden yellow setulose anterodorsally and posterodorsally, widely bare medially and ventrally. Anepimeron golden yellow setulose. Katepisternum with patch of white setulae dorsally and a few white setulae ventrally. Katatergite long microtrichose, anatergite short microtrichose. Calypter and halter yellowish white.

Wing: hyaline; microtrichose, except bare on basal 1/3 of cell r_1_, most of cell br (only microtrichose along vena spuria and in apical 1/5), posterobasal 2/3 of cell bm, anterobasal 1/3 of cell cup, and basomedian 1/2 of alula.

Legs: brown, with femora, tibiae around cicatrices, and basal tarsomeres a bit darker than other parts; yellow and white setulose. Coxae and trochanters brown; yellow and white setulose.

***Abdomen*.** Tergites dark brown, except lateral margins pale brown, and tergite 4 with two large yellowish brown maculae on posterior 2/5, which are connected along posterior margin. Tergite 1 yellowish white setulose. Tergite 2 shiny; yellowish white setulose. Tergite 3 semi-shiny with dark and dull, characteristically shaped macula over most of width; yellowish white setulose on shiny parts, black setulose on dull part. Tergite 4 semi-shiny; yellowish white setulose laterally and posteriorly, black setulose anteriorly and medially. Sternites 1–3 dark brown; yellowish white setulose. Sternite 4 yellowish brown; dark brown setulose. Genitalia as in Fig. 143.

**Female.** Unknown.

##### Diagnosis.

**Figures 76–81.**
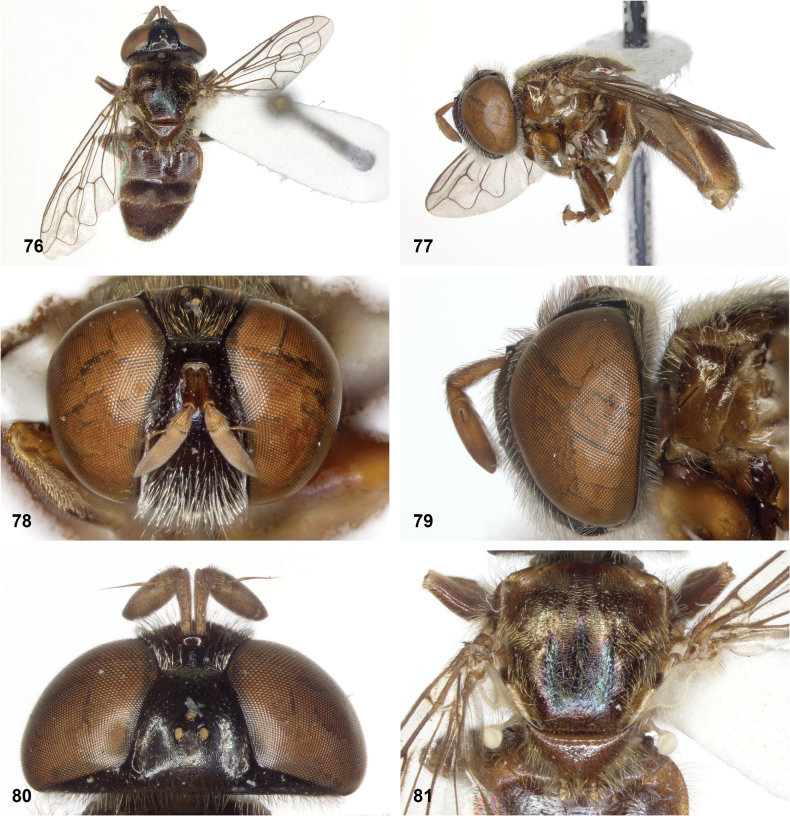
*Serichlamysmellimitis* Reemer, sp. nov. male, holotype **76** habitus, dorsal **77** habitus, lateral **78** head, frontal **79** head, lateral **80** head, lateral **81** thorax, dorsal.

Body length: male 5.5–6.5 mm (*n* = 4). Together with *S.melamitis* Reemer, sp. nov. and *S.mitis*, this species combines a black face with antennae in which the postpedicel has a dark tip (Figs 78, 79). This species differs from the other two by the characteristically shaped dull macula on tergite 3, which is wide and has three posterior ‘lobes’ or extensions (Figs 14, 76). It also differs from the other two species by the widely yellow lateral margins of tergites 2–4 (Figs 76, 77). Male genitalia as in Fig. 143.

##### Etymology.

The species name refers to the species *Serichlamysmitis* (Curran), to which this new species is very similar. The prefix *melli* (from the Latin *mellis*, honey) was chosen because this species is more honey-coloured than *S.mitis*.

##### Distribution.

This species is only known from Belo Horizonte in the Brazilian State of Minas Gerais.

#### ﻿ Serichlamys mitis

Taxon classificationAnimaliaDipteraSyrphidae

(Curran, 1940)

636A7997-6D09-50D0-9718-649559594F67

Figs 8 , 10 , 15 , 16 , 19 , 21 , 23 , 82–86 , 144

Microdon mitis Curran, 1940: 7. Holotype ♂: Brazil, Rio de Janeiro (AMNH). [examined]
Microdon mitis Curran: Thompson et al. 1976: 66.
Serichlamys mitis (Curran): Reemer and Ståhls 2013a: 148.

##### Studied type specimens.

Brazil • 1 ♂, holotype of *Microdonmitis* Curran; Rio de Janeiro; Dist. Federal; Sept. 1938; Servico Febre Amarela leg.; AMNH • 1 ♂, paratype of *Microdonmitis* Curran; Rio de Janeiro; Dist. Federal; Sept. 1938; Servico Febre Amarela leg.; USNM.

##### Additional specimens.

Brazil • 2 ♂ 1 ♀; Rio de Janeiro, Dist. Federal; Sept. 1938; Servico Febre Amarela leg.; ZMUC • 19 ♂ 7 ♀; Rio de Janeiro, Dist. Federal; Sept. 1938; Servico Febre Amarela leg.; USNM • 1 ♂; Rio de Janeiro, Dist. Federal; Sept. 1938; Servico Febre Amarela leg.; RMNH [M. Reemer specimen code MR0172].

##### Diagnosis.

Body length: male 6.5–8 mm (*n* = 24), female 7.5–9.5 mm (*n* = 8). Together with *S.melamitis* Reemer, sp. nov. and *S.mellimitis* Reemer, sp. nov., this species combines a black face with antennae in which the postpedicel has a dark tip. This species differs from *S.melamitis* Reemer, sp. nov. by colouration of the tarsi (brown with apical tarsomere yellow, instead of entirely black) (Fig. 19). The male differs from *S.melamitis* Reemer, sp. nov. in the presence of a median projection on the ventral lobe of the surstylus (Fig. 144). The female differs from *S.melamitis* Reemer, sp. nov. in the brown scutellum (yellow in *S.melamitis* Reemer, sp. nov.).

**Figures 82–86.**
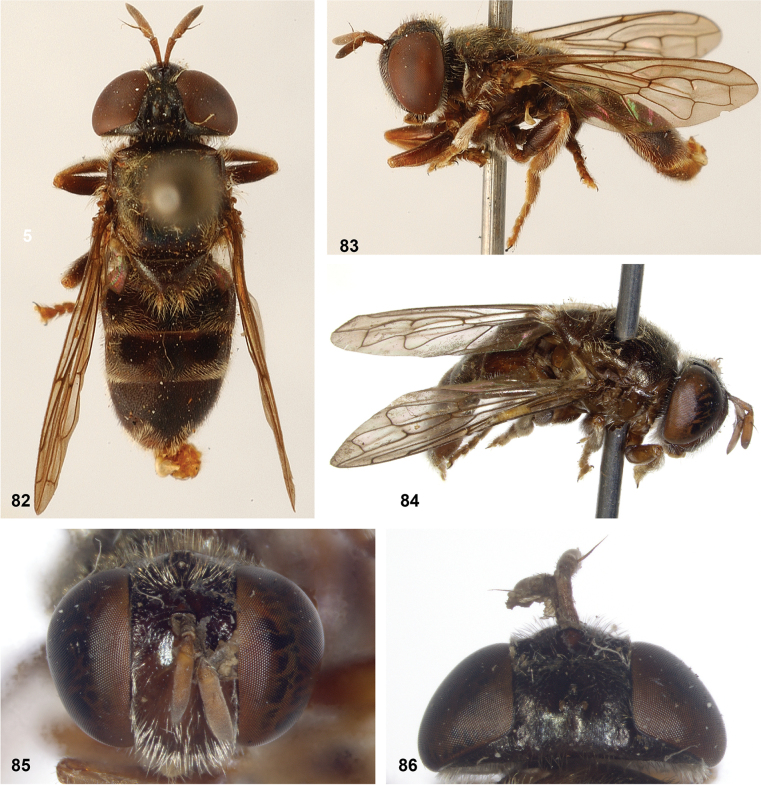
**82, 83***Serichlamysmitis* (Curran, 1940) male, holotype **82** habitus, dorsal **83** habitus, lateral **84–86***Serichlamysmitis* (Curran, 1940) female, additional specimen USNMENT01866931**84** habitus, dorsolateral **85** head, frontal **86** head, dorsal.

From *S.mellimitis* Reemer, sp. nov. this species differs in the presence of three separate dull maculae on tergite 3 (Figs 15, 16), and the brown lateral margins of the tergites (Figs 82–84). Male genitalia as in Fig. 144.

##### Distribution.

This species is only known from Rio de Janeiro in Brazil.

#### ﻿ Serichlamys mus

Taxon classificationAnimaliaDipteraSyrphidae

(Curran, 1936)

F78BF59C-98B3-5060-B3D3-14B82DD2A7F8

Figs 37 , 40 , 44 , 45 , 50 , 87–92 , 146

Microdon mus Curran, 1936: 5. Holotype ♂: Brazil, São Paulo (AMNH). [examined]
Microdon mus Curran: Thompson et al. 1976: 66.
Serichlamys mus (Curran): Reemer and Ståhls 2013a: 148.

##### Studied type specimens.

Brazil • 1 ♂, holotype of *Microdonmus* Curran; Juiquia, São Paulo; Sep. 1929; J. Lane leg.; AMNH. Label 1: “Juquia-S.P. // J. Lane, XI, 1929”; label 2 (red): “Microdon // mus [male symbol] // Curran // Holotype”; label 3: “Microdon // mus // Curran”.

**Figures 87–92.**
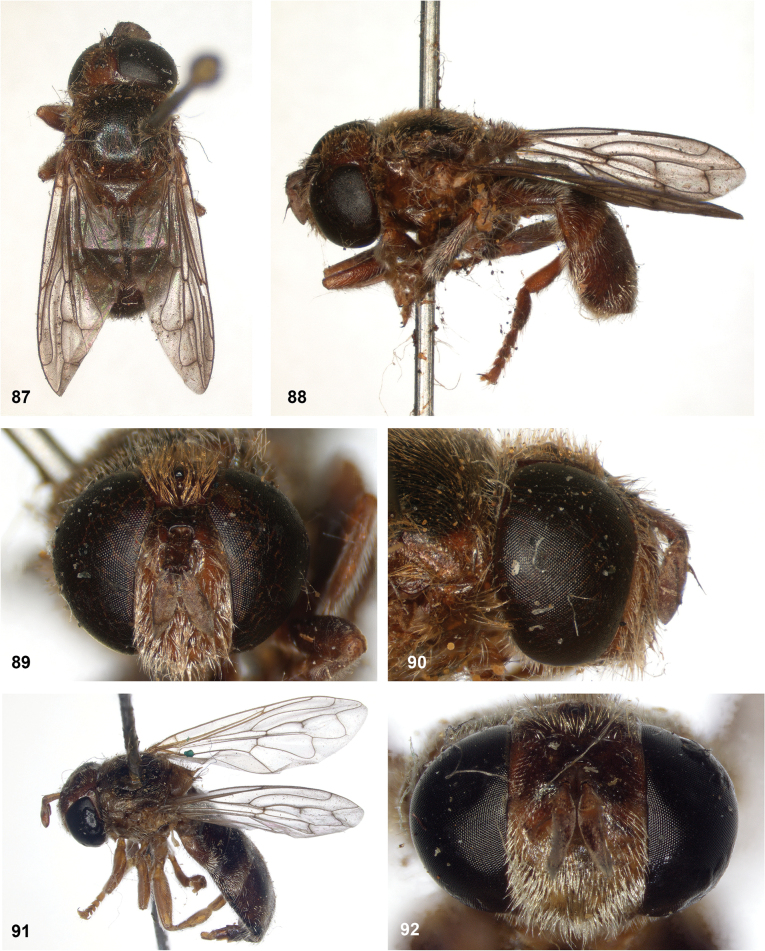
**87–90***Serichlamysmus* (Curran, 1936) male, holotype **87** habitus, dorsal **88** habitus, lateral **89** head, frontal **90** head, lateral **91, 92***Serichlamysmus* (Curran, 1936) female (Salesópolis, coll. MZUSP) **91** habitus, lateral **92** head, frontal.

##### Additional specimens.

Brazil • 1 ♂; Angra dos Reis, Jopuhyba [?] F. do Rio; Trav. et Lopes leg.; USNM [USNM specimen barcodes: USNMENT01866933; M. Reemer specimen code MR1587] • 1 ♀; São Paulo, Salesópolis, Est. Biol. Boraceia; 18 Oct. 1960; K. Lenko leg.; MZUSP.

##### Diagnosis.

Body length: male 7–7.5 mm (*n* = 2), female: 9 mm (*n* = 1). Among the species of *Serichlamys* with a yellow face, this species can be identified by the following combination of characters: abdomen with greatest width at transition of tergites 2 and 3, scutellar calcars acute (not flattened or spoon-shaped), antennae pale brown, legs entirely brown with femora only slightly darker than tibiae. Male genitalia as in Fig. 146. The female cannot be distinguished with certainty from specimens of *S.serpentiphallus* Reemer, sp. nov. and *S.simpliciphallus* Reemer, sp. nov.

##### Distribution.

This species is known from the Brazilian states São Paulo and Rio de Janeiro.

#### ﻿ Serichlamys pallitarsis

Taxon classificationAnimaliaDipteraSyrphidae

Reemer & Mengual sp. nov.

D5C51706-9320-51F5-A0E7-2C69CD12DCAC

https://zoobank.org/B8319CB1-EAA5-461D-B10C-7689334FCD8E

Figs 9 , 28 , 32 , 34 , 93–97 , 140

##### Type material.

***Holotype*.** Colombia • 1 ♂, holotype of *Serichlamyspallitarsis* sp. nov.; Valle de Cauca, San Antonio; 3°29.14'N, 76°37.60'W; 24 Feb. 2006; B.J. & F.C. Thompson leg; USNM. Label 1: “Colombia. Valle de Cauca // San Antonio // 76 37.60 W 3 29.14 N // 24 Feb 2006 // BL & FC Thompson”; USNM barcode label: USNMENT00035809. ***Paratypes*.** Colombia • 2 ♂ 1 ♀ with same data as holotype ; USNM • 1 ♂; Caldas Manizales, Corregimiento, La Palmas, Parque Rio Blanco; 5°4.98'N, 75°25.07'W; 18 Feb. 2006; B.J. & F.C. Thompson leg.; USNM • 1 ♂; Dept. Valle del Cauca, Cali, Cerro San Antonio; 15 Feb. 2006; 2200 m asl; X. Mengual leg.; ZFMK; DNA voucher code MZH:Y370; GenBank accession no. PQ629012 • 1 ♂; Dept. Valle del Cauca, Cali, Cerro San Antonio; 24 Feb. 2006; 2200 m asl; X. Mengual leg.; ZFMK; DNA voucher code MZH:Y369; GenBank accession no. HF569344 • 1 ♂; Saladito, Vallé; 2000 m asl; 17 Feb. 1970; D.M. Wood leg.; CNC.

**Figures 93–97.**
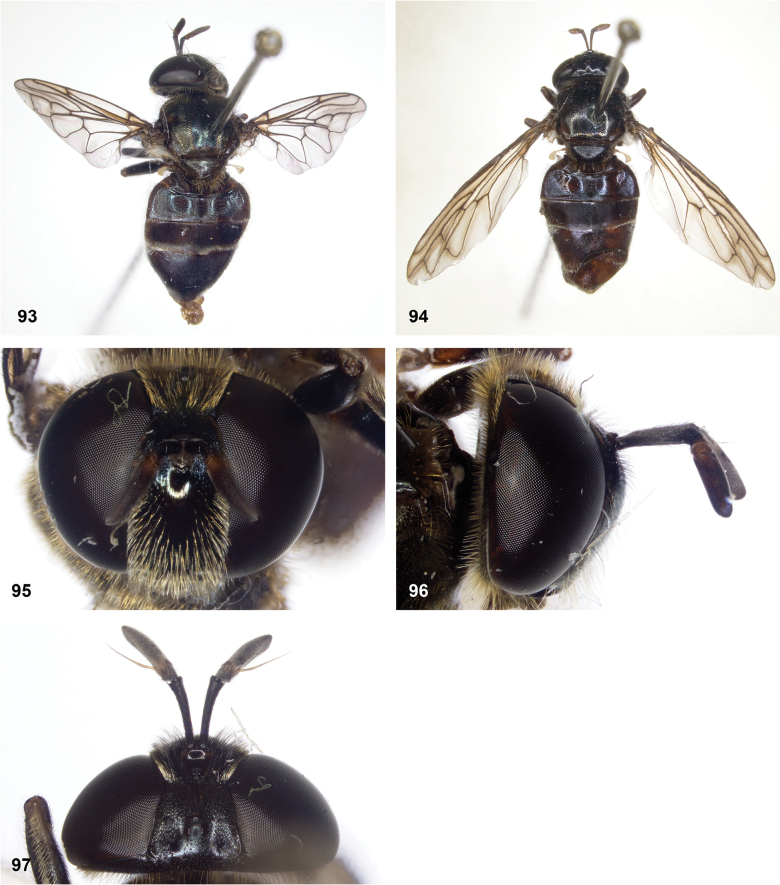
*Serichlamyspallitarsis* Reemer & Mengual, sp. nov. male, holotype (except S34: female, paratype) **93** habitus, dorsal **94** habitus, dorsal (female) **95** head, frontal **96** head, lateral **97** head, dorsal.

##### Additional specimens.

Colombia • 1 ♂; Pichindé, Vallé; 1800 m asl; 24 Feb. 1970; D.M. Wood leg.; CNC • 1 ♀; W. Colombia, Villa Karolina; 1600 m asl; 10 Jul. 1908; NHMUK [C.T. Trechmann Bequest, B.M. 1964-549].

##### Description

**(based on holotype). Adult male** Body size: 9 mm.

***Head*.** Face occupying ~ 2/5 of head width in frontal view; shiny black; yellowish white setulose, except black setulose on dorsal 1/4. Gena very narrow; black; white setulose. Oral margin laterally not produced. Frons black; medially bare, laterally black setulose except yellowish white setulose along eye margin. Vertex black; golden yellow setulose. Occiput black; dorsal 1/2 golden yellow setulose, ventral 1/2 white setulose. Eye bare. Antenna: scape black, pedicel and postpedicel dark brown; antennal ratio as ~ 7:1:7.

***Thorax*.** Scutum shiny black with faint greenish metallic sheen, margins shiny dark brown; golden yellow setulose. Postpronotum and postalar callus shiny dark brown; golden yellow setulose. Scutellum trapezoid, shiny dark brown, with two apical calcars as long as ~ 1/3 of scutellar length. Pleura shiny brown, except meron and ventral parts of katepisternum blackish. Anepisternum with shallow sulcus; golden yellow setulose anterodorsally and posterodorsally, widely bare medially and ventrally. Anepimeron golden yellow setulose. Katepisternum with patch of yellowish setulae dorsally and a few yellowish setulae ventrally. Katatergite long microtrichose, anatergite short microtrichose. Calypter and halter pale yellow.

Wing: hyaline; microtrichose, except bare on basal 2/5 of cell r_1_, most of cell br (only microtrichose along vena spuria), posterobasal 1/2 of cell bm, anterobasal 1/2 of cell cup, and basomedian 4/5 of alula.

Legs (Figs 9, 28): femora blackish brown, with apices narrowly yellowish; black setulose anteriorly, yellow setulose posteriorly. Tibiae dark brown with vaguely demarcated blackish rings; yellow and white setulose. Tarsi yellowish brown; black setulose dorsally, yellow setulose ventrally. Coxae and trochanters blackish; yellow and white setulose.

***Abdomen*.** Tergites blackish, with lateral and posterior margins brown. Tergite 1 yellowish setulose. Tergite 2 shiny (subshiny on lateral 1/4 due to microsculpture) with three dull maculae: a small oval median one and a pair of small oblique lateral ones; yellowish setulose laterally, white setulose medially. Tergite 3 dull on most of surface, with lateral and posterior margins shiny; yellow setulose on shiny parts, black setulose on dull parts. Tergite 4 semi-shiny; golden yellow setulose laterally and posteriorly, black setulose anteriorly and medially. Sternites brown; yellow setulose. Genitalia as in Fig. 140.

**Female.** As male, except for following differences. Face, frons, vertex, and occiput entirely black setulose. Postpedicel orange. Scutum, scutellum, and pleura black setulose. Wings infuscate, especially around veins. Tergite 5 yellowish brown.

##### Diagnosis.

Body length: male 8.5–10 mm (*n* = 4), female 11 mm (*n* = 1). Superficially, this species looks most similar to *S.boti* Reemer, sp. nov., *S.chloraspis* Reemer, sp. nov., and *S.varicaudata* Reemer & Mengual, sp. nov., which are of similar size and colouration. These species also share the following combination of characters: face black, tibiae entirely brown, tergite 3 broadly dull with narrow shiny margins. From *S.chloraspis* Reemer, sp. nov. and *S.varicaudata* Reemer & Mengual, sp. nov. this species differs by the entirely orange tarsi (dark in *S.chloraspis* Reemer, sp. nov. and *S.varicaudata* Reemer & Mengual, sp. nov.). From *S.boti* Reemer, sp. nov. it differs by the entirely microtrichose wing cell dm (partly bare in *S.boti* Reemer, sp. nov.), as well as by the presence of 4 or 5 basoventral setae on the surstylus (2 in *S.boti* Reemer, sp. nov.). Male genitalia as in Fig. 140.

##### Etymology.

The specific epithet is a noun referring to the pale (*pallidus*, L. = pale) tarsi of this species.

##### Molecular data.

The two paratype males from Cerro San Antonio were successfully sequenced and cluster together in our NJ tree (BS = 100). The obtained DNA barcodes are short (396 bp and 431 bp in length) and have a 99.468% similarity. The DNA barcodes of this species show affinity to those of *S.varicaudata* Reemer & Mengual, sp. nov. (similarity = 92.235–94.382%).

##### Distribution.

This species is only known from the Valle del Cauca area on the western slopes of the Andes in Colombia.

#### ﻿ Serichlamys serpentiphallus

Taxon classificationAnimaliaDipteraSyrphidae

Reemer sp. nov.

3F8873C6-4097-5259-A662-6D7B6130E909

https://zoobank.org/DC250489-16B0-400D-AADB-69E5874A0C0E

Figs 6 , 42 , 48 , 53–55 , 98–102 , 148

##### Type material.

***Holotype*.** Brazil • 1 ♂, holotype of *Serichlamysserpentiphallus* sp. nov.; Nova Teutonia; 27°11'S, 52°23'W; Oct. 1968; 300–500 m asl; F. Plaumann leg; RMNH. Label 1: “Brasilien // Nova Teutonia // 27° 11 B, 52° 23 L // Fritz Plaumann // X.1968 // 300–500 m”; label 2: “Museum Leiden // Collectie // Van Doesburg // rec. 1973”; label 3: “Microdon // mus Curr. // det. v. Doesburg”; label 4: “Serichlamys sp. // Det. M. Reemer 2022 // Specimen code MR1486”; label 5 (red): “HOLOTYPE // Serichlamys // serpentiphallus // M. Reemer”. ***Paratypes*.** Brazil • 6 ♂ with same data as holotype; RMNH • 1 ♂ with same data as holotype except date Oct. 1967; RMNH • 1 ♂ with same label data as holotype except date Nov. 1971; USNM [USNMENT01866947 • 1 ♂; Itatiaya, Est. Biologica; 24 Oct. 1932; 1100 m asl; E. de Rio leg.; MZUSP.

**Figures 98–102.**
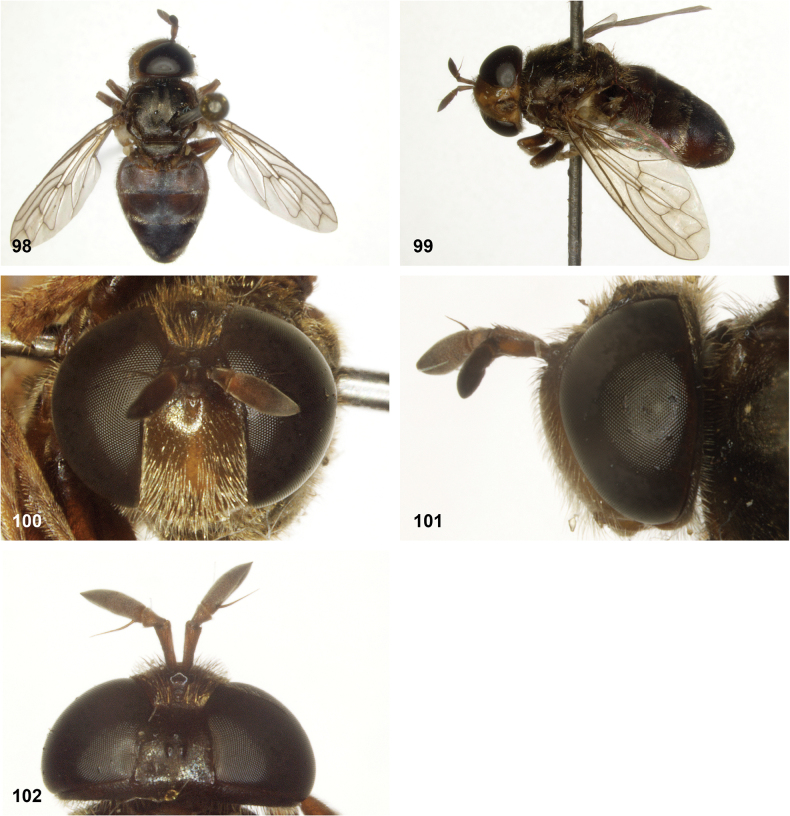
*Serichlamysserpentiphallus* Reemer, sp. nov. male, holotype **98** habitus, dorsal **99** habitus, lateral **100** head, frontal **101** head, lateral **102** head, dorsal.

##### Additional specimens.

Brazil • 1 ♂; Nova Teutonia; 27° 11’ B, 52° 23’ L; 300–500 m asl; Nov. 1964; RMNH [M. Reemer specimen code: MR0381] • 1 ♂; São Paulo, São Luis do Paraitinga, PESM, Núcleo Sta. Virginia; 23.324194°S, 45.094000°W; 20 Nov. 2011; N.W. Perioto leg.; CNC [CNC specimen code: CNC1059094] • 1 ♀; São Paulo, São Luis do Paraitinga, PESM, Núcleo Sta. Virginia; 23.324194°S, 45.094000°W; 20 Nov. 2011; N.W. Perioto leg.; CNC [CNC specimen code: CNC1059095]; GenBank accession no. PQ629021.

##### Description

**(based on holotype). Adult male** Body size: 8 mm.

***Head*.** Face occupying ~ 1/3 of head width in frontal view; shiny yellowish brown (in Fig. 100 there appears to be a yellow median vitta, but this is an artefact of lighting conditions, which highlight some pale subcuticular structure); yellowish white setulose. Gena yellow; white setulose. Oral margin laterally hardly produced. Frons brown; medially bare, laterally black setulose. Vertex brown; golden yellow setulose. Occiput brown; dorsal 1/2 golden yellow setulose, ventral 1/2 white setulose. Eye bare. Antenna orange-brown; antennal ratio ~ 2:1:3.

***Thorax*.** Scutum blackish brown with bronze sheen, margins brown; golden yellow setulose. Postpronotum and postalar callus brown; golden yellow setulose. Scutellum trapezoid, brown; golden yellow setulose; with two dorsoventrally flattened, spoon-shaped calcars as long as ~ 1/3 of scutellar length. Pleura brown. Anepisternum with shallow sulcus; golden yellow setulose anterodorsally and posterodorsally, widely bare medially and ventrally. Anepimeron golden yellow setulose. Katepisternum with patch of white setulae dorsally and a few white setulae ventrally. Katatergite long microtrichose, anatergite short microtrichose. Calypter and halter pale yellow.

Wing: hyaline; microtrichose, except bare on narrow strip along vein RS in cell r_1_, most of cell br (only microtrichose along vena spuria), posterobasal 1/5 of cell bm, anterobasal 1/4 of cell cup, and basomedian 2/3 of alula.

Legs: pale brown, with femora and hind metatarsus somewhat darker; yellow to white setulose, except mid and hind tarsus dorsally with some black setulae. Coxae and trochanters brown; yellow to white setulose.

***Abdomen*.** Tergites dark brown, except lateral margins and posterior margin of tergite 4 paler brown. Tergite 1 white setulose. Tergite 2 medially shiny except for small median patch of grey microtrichia, laterally dull due to grey microtrichia; medially white setulose, laterally yellow setulose. Tergite 3 dull on most of surface, with lateral and posterior margins shiny; black setulose on dull parts, yellowish white setulose on shiny parts. Tergite 4 semi-shiny; golden yellow setulose laterally and posteriorly, black setulose anteriorly and medially. Sternites yellowish brown; yellowish white setulose. Genitalia as in Fig. 148.

**Female.** One female probably belonging to this species was collected at the same site and date as one of the males listed among the additional specimens. It differs in paler overall colouration, which is probably either a result of the specimen being teneral at time of collecting, or of preservation methods. Tergites 4 and 5 are entirely yellowish brown.

##### Diagnosis.

Body length: male 7.5–8.5 mm (*n* = 10), female 8.5 mm (*n* = 1). Together with *S.simpliciphallus* Reemer, sp. nov. and *S.spathulata* Reemer, sp. nov., this species belongs to a group of three species of which most (not all!) specimens have dorsoventrally flattened, spoon-shaped calcars on the scutellum (Figs 51–57). From *S.spathulata* Reemer, sp. nov. it differs by the presence of separate patches of microtrichia on tergite 2 (Fig. 48), the brown postpedicel, and the structure of the male genitalia (Fig. 148). From *S.simpliciphallus* Reemer, sp. nov. this species can only be reliably distinguished by the structure of the male genitalia, most notably the shape of the phallus: S-shaped in *S.serpentiphallus* Reemer, sp. nov., straight in *S.simpliciphallus* Reemer, sp. nov. Other characters, such as colouration of integument and setulosity, as well as distribution of microtrichia on the tergites, were found to be too variable among the studied specimens. Females are undistinguishable from *S.simpliciphallus* Reemer, sp. nov. at present.

##### Etymology.

The name *serpentiphallus* is a noun composed of the Latin words *serpens* (snake) and *phallus* (phallus). It refers to the s-shaped phallus of the male of this species.

##### Molecular data.

The female from São Luis do Paraitinga (CNC1059095) was successfully sequenced. The identification of this specimen was based on the fact that it was collected at the same locality and date as CNC1059094, a male which was identified based on its genitalia. The DNA barcode is placed with high support (BS = 99.6) together with a cluster that includes the sequences of *S.spathulata* Reemer, sp. nov. and *Serichlamys* sp. (CNC1059093; GenBank accession no. PQ629019), although they differ considerably (similarity = 87.459–87.541%)

##### Distribution.

This species is known from the Brazilian States of São Paulo and Rio de Janeiro.

#### ﻿ Serichlamys simpliciphallus

Taxon classificationAnimaliaDipteraSyrphidae

Reemer sp. nov.

4CF2093C-EBB3-5962-9928-F7A395FF357F

https://zoobank.org/12E93A25-81BE-4094-9978-067E39480675

Figs 39 , 43 , 56 , 57 , 103–106 , 149

##### Type material.

***Holotype*.** Brazil • 1 ♂, holotype of *Serichlamyssimpliciphallus* sp. nov.; São Paulo, Campos de Jordão; 28 Nov. 1957; K. Lenko leg.; MZUSP. Label 1: “Campos de Jordão // S. Pãulo BRASIL // 28.XI.1957 // K. Lenko leg.”; label 2: “Serichlamys sp. // Det. M. Reemer 2024 // Specimen code MR1588”. ***Paratypes*.** Brazil • 1 ♂; São Paulo, Campos de Jordão; 19 Nov. 1957; K. Lenko leg.; MZUSP • 4 ♂; #; São Paulo, Campos de Jordão; 25 Nov. 1957; K. Lenko leg.; MZUSP • 1 ♂; Campos de Jordão; 26 Nov. 1957; K. Lenko leg.; MZUSP • 1 ♂; Campos de Jordão; 27 Nov. 1957; K. Lenko leg.; MZUSP • 3 ♂; Campos de Jordão; 28 Nov. 1957; K. Lenko leg.; MZUSP • 1 ♂; Campos de Jordão; 28 Nov. 1957; K. Lenko leg.; RMNH • 1 ♂; Itatiaya, Macieiras; 1960 m asl; 11 Nov. 1933; J.F. Zikán leg; MZUSP.

**Figures 103–106.**
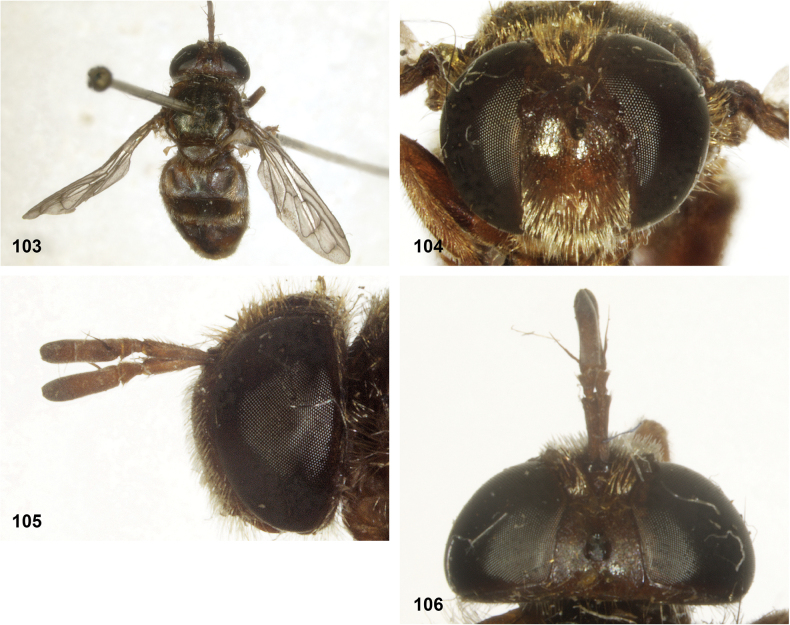
*Serichlamyssimpliciphallus* Reemer, sp. nov. male, holotype **103** habitus, dorsal **104** head, frontal **105** head, lateral **106** head, frontal.

##### Additional specimens.

Brazil • 1 ♀; São Paulo, Campos de Jordão; 28 Nov. 1957; K. Lenko leg.; MZUSP.

##### Description

**(based on holotype). Adult male** Body size: 8 mm.

***Head*.** Face occupying ~ 1/3 of head width in frontal view; shiny yellowish brown; white setulose. Gena yellow; white setulose. Oral margin laterally hardly produced. Frons brown; medially bare, laterally golden yellow setulose. Vertex brown; golden yellow setulose. Occiput brown except medially blackish; dorsal 1/2 golden yellow setulose, ventral 1/2 white setulose. Eye bare. Antenna orange-brown; antennal ratio as ~ 4:1:4.

***Thorax*.** Scutum blackish with bronze sheen, margins brown; golden yellow setulose. Postpronotum and postalar callus brown; golden yellow setulose. Scutellum trapezoid, brown; golden yellow setulose; with two dorsoventrally flattened, spoon-shaped calcars as long as ~ 1/4 of scutellar length. Pleura brown. Anepisternum with shallow sulcus; golden yellow setulose anterodorsally and posterodorsally, widely bare medially and ventrally. Anepimeron golden yellow setulose. Katepisternum with patch of white setulae dorsally and a few white setulae ventrally. Katatergite long microtrichose, anatergite short microtrichose. Calypter and halter pale yellow.

Wing: hyaline; microtrichose, except bare on most of cell br (only microtrichose along vena spuria), posterobasal 1/5 of cell bm, anterobasal 1/5 of cell cup, and basomedian 2/5 of alula.

Legs: pale brown, with femora and hind metatarsus somewhat darker; yellow to white setulose. Coxae and trochanters brown; yellow to white setulose.

***Abdomen*.** Tergites dark brown, except lateral margins and posterior margins of tergites 3 and 4 paler brown. Tergite 1 yellowish setulose. Tergite 2 with three large patches of grey microtrichiae, with narrow shiny parts in between; medially white setulose, laterally yellow setulose. Tergite 3 dull on most of surface, with lateral and posterior margins shiny; black setulose on dull parts, yellowish white setulose on shiny parts. Tergite 4 semi-shiny; golden yellow setulose laterally and posteriorly, black setulose anteriorly and medially. Sternites yellowish brown; yellowish white setulose. Genitalia as in Fig. 149.

**Female.** One female (see Additional specimens) probably belonging to this species was collected at the same site and date as one of the male paratypes. Apart from usual sexual dimorphism, no important differences were noted.

##### Diagnosis.

Body length: male 8–8.5 mm (*n* = 12), female 9 mm (*n* = 1). Together with *S.serpentiphallus* Reemer, sp. nov. and *S.spathulata* Reemer, sp. nov., this species belongs to a group of three species of which most (not all!) specimens have dorsoventrally flattened, spoon-shaped calcars on the scutellum (Figs 51–57). From *S.spathulata* Reemer, sp. nov. it differs by presence of separate patches of microtrichia on tergite 2 (Fig. 48), the brown postpedicel, and the structure of the male genitalia (Fig. 149). From *S.simpliciphallus* Reemer, sp. nov. this species can only be reliably distinguished by the structure of the male genitalia, most notably the shape of the phallus: S-shaped in *S.serpentiphallus* Reemer, sp. nov., straight in *S.simpliciphallus* Reemer, sp. nov. Other characters, such as colouration of integument and setulosity, as well as distribution of microtrichia on the tergites, were found to be too variable among the studied specimens. Females are undistinguishable from *S.serpentiphallus* Reemer, sp. nov. at present.

##### Molecular data.

An unidentified female specimen from Estação Biológica de Boracéia (Salesópolis, Sao Paulo, Brazil; CNC1059093; GenBank PQ629019) possibly belongs to *S.simpliciphallus*. Its DNA barcode clusters with the sequences of *S.spathulata* Reemer, sp. nov. (BS = 100).

##### Etymology.

The name *simpliciphallus* is a noun composed of the Latin words *simplex* (simplicity) and *phallus* (phallus). It refers to the ‘simple’ shaped phallus of the male of this species, in contrast with the otherwise morphologically very similar *S.serpentiphallus*.

##### Distribution.

This species is only known from the Brazilian State of São Paulo.

#### ﻿ Serichlamys spathulata

Taxon classificationAnimaliaDipteraSyrphidae

Reemer sp. nov.

18325362-0AEB-5D2C-BD5B-963BE2084F3E

https://zoobank.org/4DDBAC1F-8703-4089-8554-DB7A0913C2EC

Figs 41 , 46 , 47 , 51 , 52 , 107–112 , 147

##### Type material.

***Holotype*.** Brazil • 1 ♂, holotype of *Serichlamysspathulata* sp. nov.; São Paulo, Salesópolis, Estação Biológica de Boracéia; 23.652778°S, 43.890833°W; 26 Nov. 2016; Amorim leg.; CNC [CNC1059091]. Label 1: “Brazil: Sao Paulo // Salésopolis, Estação // Biológica de Boracéia base // 23.652778° S 43.890833° W // 26.xi.2016. Amorim & eq // coll. Malaise trap // CNC1059091”; GenBank accession no. PQ629007. ***Paratypes*.** Brazil • 3 ♂ with same label data as holotype except CNC specimen codes as follows: CNC1059088, CNC1059089, CNC1059090; GenBank accession no. PQ629010, PQ629014, PQ629003 • 1 ♂; Paraná, S. José Pinhais, Ser. Mar. Br 277 Km54; 24 Nov. 1986; Lev. Ent. Profaupar leg.; JTS [M. Reemer specimen code MR598] • 1 ♂; São Paulo, Campos do Jordão; Dec. 1945; MZUSP • 3 ♂ 1 ♀; São Paulo, Campos do Jordão; Dec. 1955; MZUSP • 1 ♂; São Paulo, Campos do Jordão; Dec. 1955; RMNH.

**Figures 107–112.**
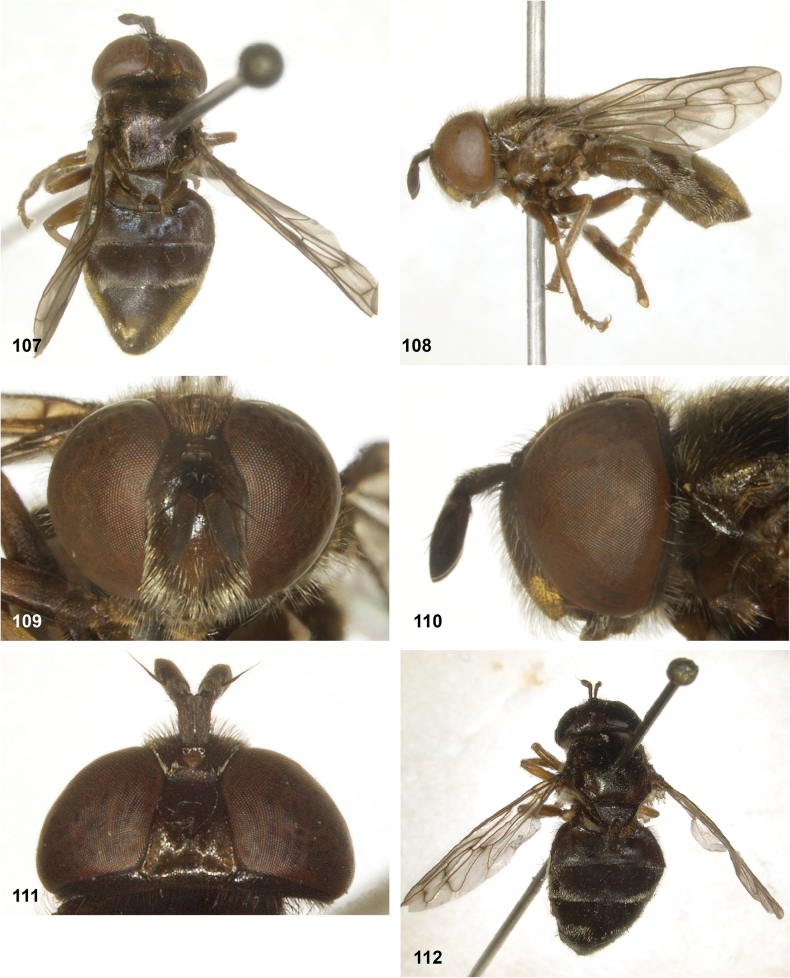
*Serichlamysspathulata* Reemer, sp. nov. male, holotype **107** habitus, dorsal **108** habitus, lateral **109** head, frontal **110** head, lateral **111** head, dorsal **112***Serichlamysspathulata* Reemer, sp. nov. female, paratype: habitus, dorsal.

##### Additional specimens.

Brazil • 1 ♀; São Paulo, São Luis do Paraitinga, PESM, Núcleo Sta. Virginia; 23.324194°S, 45.094000°W; 20 Nov. 2011; N.W. Perioto leg.; Malaise trap; CNC [CNC specimen code: CNC1059092]; GenBank accession no. PQ629006.

##### Description

**(based on holotype). Adult male** Body size: 7 mm.

***Head*.** Face occupying ~ 1/3 of head width in frontal view; shiny yellowish brown; white setulose. Gena yellow; white setulose. Oral margin laterally hardly produced. Frons brown; medially bare, laterally black setulose. Vertex brown; golden yellow setulose. Occiput brown except medially blackish; dorsal 1/2 golden yellow setulose, ventral 1/2 white setulose. Eye bare. Antenna blackish brown, basal 1/4 of postpedicel paler; antennal ratio ~ 3:1:4.5.

***Thorax*.** Scutum blackish with bronze sheen, margins brown; golden yellow setulose. Postpronotum and postalar callus brown; golden yellow setulose. Scutellum trapezoid, brown; golden yellow setulose; with two dorsoventrally flattened, spoon-shaped calcars as long as ~ 1/3 of scutellar length. Pleura brown. Anepisternum with shallow sulcus; golden yellow setulose anterodorsally and posterodorsally, widely bare medially and ventrally. Anepimeron golden yellow setulose. Katepisternum with patch of white setulae dorsally and a few white setulae ventrally. Katatergite long microtrichose, anatergite short microtrichose. Calypter and halter pale yellow.

Wing: hyaline; microtrichose, except bare on narrow strip in cell r_1_ along vein RS, most of cell br (only microtrichose along vena spuria), very narrowly along posterior margin of cell bm, and basomedian 1/3 of alula.

Legs: pale brown, with femora somewhat darker; yellow to white setulose, except mid and hind femora anteriorly black setulose. Coxae and trochanters brown; yellow to white setulose.

***Abdomen*.** Tergites dark brown, except lateral margins and posterior margins of tergites 3 and 4 paler brown. Tergite 1 white setulose. Tergite 2 largely grey microtrichose, with submedian pair of small, vaguely demarcated shiny maculae; white setulose. Tergite 3 dull on most of surface, with lateral and posterior margins shiny; black setulose on dull parts, white setulose on shiny parts. Tergite 4 semi-shiny; yellowish setulose laterally and posteriorly, black setulose anteriorly and medially. Sternites yellowish brown; white setulose. Genitalia as in Fig. 147.

**Female.** As male, except for following differences. Body length 10 mm (*n* = 1). Antenna: scape and pedicel brow, postpedicel blackish. Legs entirely yellowish brown. Tergite 5 dark brown; yellowish setulose laterally and posteriorly, black setulose anteriorly and medially.

##### Diagnosis.

Body length: male 7–8 mm (*n* = 10), female 10 mm (*n* = 1). Together with *S.serpentiphallus* Reemer, sp. nov. and *S.simpliciphallus* Reemer, sp. nov., this species belongs to a group of three species of which most (not all!) specimens have dorsoventrally flattened, spoon-shaped calcars on the scutellum (Figs 51–57). From both species, as well as from *S.mus* (Curran), it differs by the wide fascia of grey microtrichia on tergite 2, with at most a pair of smaller round bare parts laterally (Figs 46, 47). It also differs from the three aforementioned species by the black postpedicel (at most basal 1/4 paler). Male genitalia as in Fig. 147.

##### Notes.

The distribution of microtrichia on the wing is very variable. For instance, in the holotype cell cup is entirely microtrichose, but in some paratypes it is partly bare. In some specimens the alula is almost entirely microtrichose, while in other ones there is a large bare patch basomedially, and intermediates occur.

##### Etymology.

The name *spathulata* is an adjective derived from *spatha*, the Latin word for spoon. It refers to the spoon-shaped scutellar calcars.

##### Molecular data.

Five DNA barcodes were obtained for this species, which cluster together in our NJ tree (BS = 100). As sister taxon we recovered *Serichlamys* sp. (CNC1059093) (similarity = 97.264–97.416%) with high support (BS = 100).

##### Distribution.

This species is only known from the Brazilian State of São Paulo.

#### ﻿ Serichlamys trigonoides

Taxon classificationAnimaliaDipteraSyrphidae

Reemer sp. nov.

8FD053E0-7DCD-5B8F-8213-7F7A6D69496E

https://zoobank.org/2AD5AD0C-571F-495B-8197-18FAF1609CA4

**Figures 113–117.**
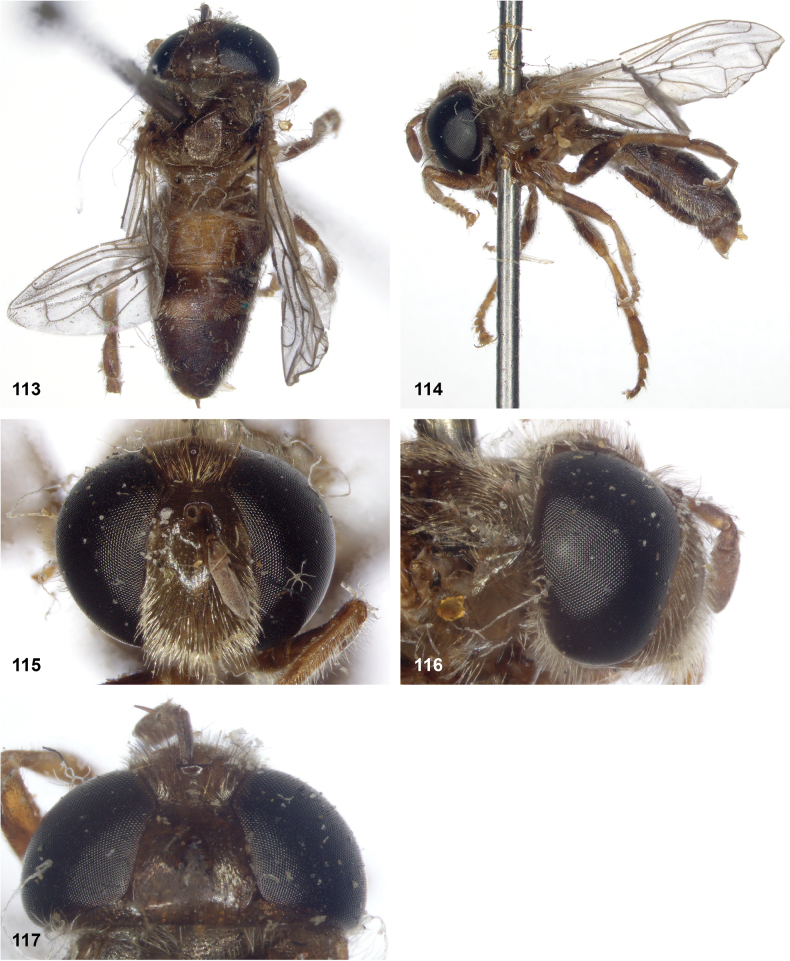
*Serichlamystrigonoides* Reemer, sp. nov. male, holotype **113** habitus, dorsal **114** habitus, lateral **115** head, frontal **116** head, lateral **117** head, dorsal.

Figs 36 , 113–117 , 145

##### Type material.

***Holotype*.** Brazil • 1 ♂, holotype of *Serichlamystrigonoides* sp. nov.; São Paulo, Praia Grande, Faz. Rondonea; Feb. 1945; M. Carrera leg; MZUSP. Label 1: “São Paulo // Praia Grande // Faz. Rondonea // Fev. – 1945 // M. Carrera coll.”; label 2: “Serichlamys sp. // Det. M. Reemer 2024 // Specimen code MR1590”.

##### Description

**(based on holotype). Adult male** Body size: 6 mm.

***Head*.** Face occupying ~ 1/3 of head width in frontal view; shiny yellowish brown; white setulose. Gena yellow; white setulose. Oral margin laterally hardly produced. Frons yellowish brown; medially bare, laterally golden yellow setulose. Vertex yellowish brown; golden yellow setulose. Occiput pale brown, white setulose. Eye bare. Antenna yellowish brown; antennal ratio ~ 2.5:1:4.5.

***Thorax*.** Scutum shiny yellowish brown; golden yellow setulose. Postpronotum and postalar callus yellowish brown; golden yellow setulose. Scutellum trapezoid, yellowish brown; black setulose medially, golden yellow setulose laterally and posteriorly; with two acute calcars as long as ~ 1/3 of scutellar length. Pleura yellowish brown. Anepisternum with shallow sulcus; golden yellow setulose anterodorsally and posterodorsally, widely bare medially and ventrally. Anepimeron golden yellow setulose. Katepisternum with patch of white setulae dorsally and a few white setulae ventrally. Katatergite long microtrichose, anatergite short microtrichose. Calypter and halter pale yellow.

Wing: hyaline; microtrichose, except bare on narrow strip in cell r_1_ along vein RS, most of cell br (only microtrichose along vena spuria), posterobasal 1/4 of cell bm, and basomedian 1/4 of alula.

Legs: pale brown, with femora and hind metatarsus somewhat darker; yellow to white setulose. Coxae and trochanters brown; yellow to white setulose.

***Abdomen*.** Tergites dark brown, except tergite 2 medially pale brown and tergite 3 with pair of wide pale brown maculae along posterior margin. Tergites 1 and 2 white setulose. Tergite 3 black setulose anteromedially, white setulose laterally and posteriorly. Tergite 4 yellowish setulose laterally and posteriorly, black setulose anteriorly and medially. Sternites yellowish; white setulose. Genitalia as in Fig. 145.

**Female.** Unknown.

##### Diagnosis.

Body length: male 6 mm (*n* = 1). This is the only known species of *Serichlamys* with a pale face in which the abdomen is at its widest around halfway tergite 2 (Figs 36, 113) (this is also the case in *S.melamitis* Reemer, sp. nov., *S.mellimitis* Reemer, sp. nov. and *S.mitis*, but in these species the face is dark). Its colouration is also characteristic, with a yellowish brown scutum and pattern of dark and pale maculae on the tergites (Fig. 36). Because of these characters, this species reminds of certain Neotropical species of stingless bees. Male genitalia as in Fig. 145.

##### Etymology.

The name *trigonoides* is an adjective derived from the name *Trigona* Jurine, 1807, a commonly occurring genus of stingless bees (Apidae: Meliponini) in South America. The habitus of *S.trigonoides* reminds of those bees.

##### Distribution.

This species is only known from the Brazilian State of São Paulo.

#### ﻿ Serichlamys varicaudata

Taxon classificationAnimaliaDipteraSyrphidae

Reemer & Mengual sp. nov.

C402A79A-34DD-5EE0-8B5C-26525CA70278

https://zoobank.org/C3620B78-3EDB-4AE8-9F47-4D049E5C804F

Figs 1–4 , 11 , 17 , 27 , 29 , 33 , 35 , 118–125 , 141

Serichlamys sp. ECU1: Wong et al. 2023: supplementary data files 1, 2, 4, 5, 7.

##### Type material.

***Holotype*.** Ecuador • 1 ♂, holotype of *Serichlamysvaricaudata* sp. nov.; Napo Prov., Yanayacu Biological station, trail up to; 0.592368°S, 77.890993°W, 2200 m asl; 18 Aug. 2022; X. Mengual leg.; INABIO. Label 1: “ECUADOR: Napo Prov., Yanayacu // Biological Station, trail up to // 0.592368° S 77.890993° W, // 2200 m., 18 Aug. 2022, hand-net // Leg.: X. Mengual”; label 2 [barcode label]: “ZFMK-DIP-00095371”. ***Paratypes*.** Ecuador • 1 ♀; Zamora Chinchipe, San Francisco, Reserva Biológica S. Francisco, trail Atajo; 03°58'30"S, 79°04'25"W; 2000 m asl; 25 Feb. – 3 Mar. 2009; M. Pollet & A. De Braekeleer leg.; yellow pan trap; RBINS • 3 ♂; Zamora-Chinchipe Prov., Cantón Zamora, Sector San Francisco, ECSF, T2 to Camino Canal; 8 Aug. 2012; X. Mengual leg.; 2 in ZFMK [ZFMK-DIP-00107320, ZFMK-DIP-00107321], 1 in INABIO [ZFMK DIP00107323] • 1 ♂ 1 ♀; Zamora-Chinchipe Prov., Cantón Zamora, Sector San Francisco, ECSF; 26 Jul. 2012; X. Mengual leg.; ZFMK [ZFMK-DIP-00107324, ZFMK-DIP-00107325] • 1 ♂; Zamora-Chinchipe Prov., Cantón Zamora, Sector San Francisco, ECSF; 31 Jul. 2012; X. Mengual leg.; ZFMK [ZFMK-DIP-00107326] • 1 ♂, Napo Prov., Yanayacu Biological Station, grassland near station; 0.598355°S, 77.891378°W; 2150 m asl; 18 Aug. 2022; X. Mengual leg.; ZFMK [ZFMK-DIP-00095358]; GenBank accession no. PQ629013 • 1 ♂, Napo Prov., Yanayacu Biological Station, grassland near station; 0.598355°S, 77.891378°W; 2150 m asl; 21 Aug. 2022; X. Mengual leg.; ZFMK [ZFMK-DIP-00095500]; GenBank accession no. PQ628993 • 2 ♂, Napo Prov., Yanayacu Biological Station, grassland near station; 0.598355°S, 77.891378°W; 2150 m asl; 22 Aug. 2022; X. Mengual leg.; ZFMK [ZFMK-DIP-00095541] and RMNH [ZFMK-DIP-00095542]; GenBank accession no. PQ629004, PQ629002.

**Figures 118–125.**
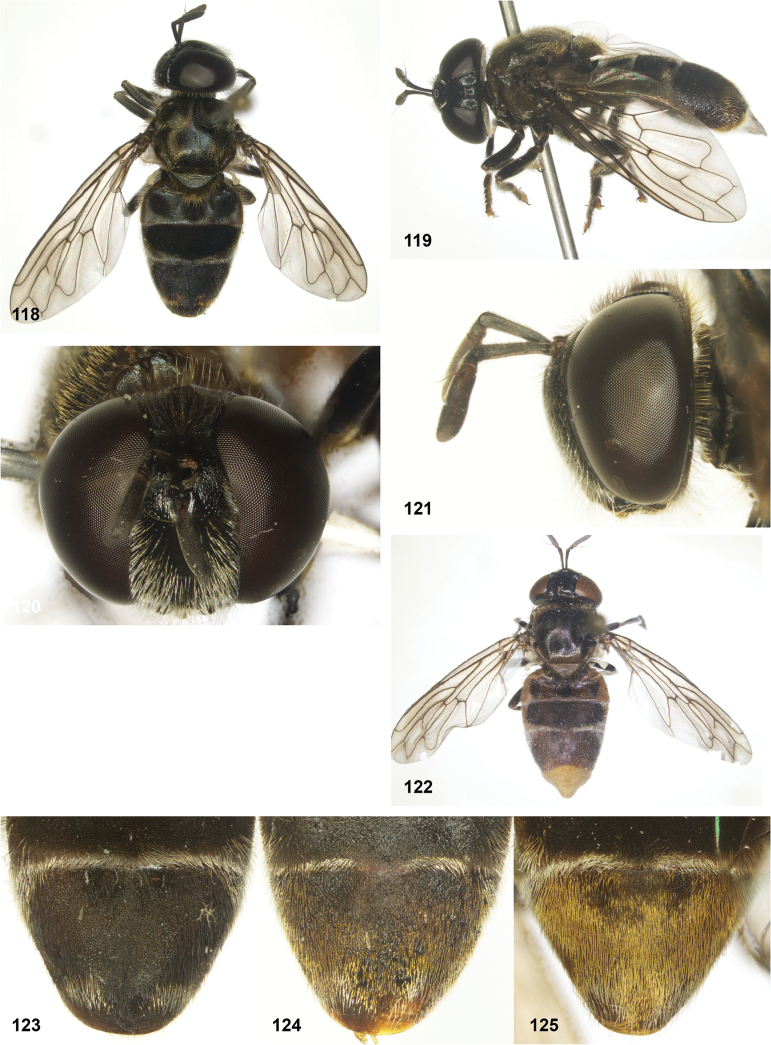
**118–121***Serichlamysvaricaudata* Reemer & Mengual, sp. nov. male, holotype **118** habitus, dorsal **119** habitus, lateral **120** head, frontal **121** head, lateral **122***Serichlamysvaricaudata* Reemer & Mengual, sp. nov. female, paratype: habitus, dorsal **123–125***Serichlamysvaricaudata* Reemer & Mengual, sp. nov. male: variation in colouration of setulosity of tergite 4.

##### Additional specimens.

Ecuador • 1 ♂; Zamora-Chinchipe Prov., Cantón Zamora, Sector San Francisco, ECSF; 26–31 Jul. 2012; X. Mengual leg.; CNC [M. Reemer specimen code MR0425, ZFMK-DIP-00107327]; GenBank accession no. PQ628994 • 3 ♂; Zamora-Chinchipe Prov., Cantón Zamora, Sector San Francisco, ECSF; 8 Aug. 2012; X. Mengual leg.; CNC [J. Skevington mol. spec. #5471, ZFMK-DIP-00107329; J. Skevington specimen 30567, GenBank accession no. PQ628997; J. Skevington specimen 30592, GenBank accession no. PQ628999] • 2 sex unknown; same metadata as previous [J. Skevington specimen 30594, GenBank accession no. PQ628998; GenBank accession no. OR183415] • 4 ♂; Zamora-Chinchipe Prov., Cantón Zamora, Sector San Francisco, ECSF; 31 Jul. – 11 Aug. 2012; 03°58'29"S, 79°04'42"W; 1890 m asl; X. Mengual leg.; ZFMK [ZFMK-DIP-00068712, 00068713, 00068716, 00068717]; GenBank accession no. PQ629005, PQ629009, PQ629020, PQ629017 • 1 ♂; Zamora-Chinchipe Prov., Cantón Zamora, Sector San Francisco, ECSF; 31 Jul. – 11 Aug. 2012; 03°58'29"S, 79°04'42"W; 1890 m asl; X. Mengual leg.; ZFMK [ZFMK-DIP-00013508]; • 2 ♀; Zamora-Chinchipe Prov., Cantón Zamora, Sector San Francisco, ECSF; 31 Jul. – 11 Aug. 2012; 03°58'29"S, 79°04'42"W; 1890 m asl; X. Mengual leg.; RMNH [ZFMK-DIP-00068714] and ZFMK [ZFMK-DIP-00068715]; GenBank accession no. PQ628995, PQ629001 • 1 ♂; Zamora-Chinchipe Prov., Cantón Zamora, Sector San Francisco, ECSF; 8 Aug. 2012; X. Mengual leg.; ZFMK [ZFMK-DIP-00068711]; GenBank accession no. PQ629011 • 1 ♂; Napo Prov., Yanayacu Biological Station; 00°35'44.3"S, 77°53'38.6"W; 2234 m asl; 29 Aug. - 3 Sep. 2019; X. Mengual leg.; ZFMK [ZFMK-DIP-00068683]; GenBank accession no. PQ629000 • 1 ♂; Napo Prov., Yanayacu Biological Station, trail up to; 0.592368°S, 77.890993°W; 2200 m asl; 18 Aug. 2022; X. Mengual leg.; ZFMK [ZFMK-DIP-00095695] • 5 ♂; Napo Prov., Yanayacu Biological Station, environs; 0.5991°S, 77.891495°W; 2140 m asl; 14 Sep. 2023; X. Mengual leg.; INABIO [ZFMK-DIP-00103503] and ZFMK [ZFMK-DIP-00103504, ZFMK-DIP-00103505, ZFMK-DIP-00103507, ZFMK-DIP-00103508] • 2 ♂; Napo Prov., Yanayacu Biological Station, stream trail; 0.59952°S, 77.89434°W; 2180 m asl; 17 Sep. 2023; X. Mengual leg.; ZFMK [ZFMK-DIP-00103600, ZFMK-DIP-00103607] • 1 ♂; Napo Prov., Yanayacu Biological Station, forest edge; 0.5991°S, 77.891495°W; 2140 m asl; 14 Sep. - 19 Sep. 2023; X. Mengual leg.; Malaise trap; ZFMK [ZFMK-DIP-00104346].

##### Description

**(based on holotype). Adult male** Body size: 9.5 mm.

***Head*.** Face occupying ~ 2/5 of head width in frontal view; shiny black; white setulose, except black setulose on dorsal 1/4. Gena very narrow; black; white setulose. Oral margin laterally not produced. Frons black; medially bare, laterally black setulose except white setulose along eye margin. Vertex black; black setulose. Occiput black; dorsal 1/2 golden yellow setulose, ventral 1/2 white setulose. Eye bare. Antenna: scape black, pedicel and postpedicel dark brown; antennal ratio ~ 4:1:5.

***Thorax*.** Scutum shiny black with bronze sheen; golden yellow setulose. Postpronotum and postalar callus shiny brown; golden yellow setulose. Scutellum trapezoid, of same colour as scutum, with two apical calcars as long as ~ 2/5 of scutellar length. Pleura greyish brown, except meron and ventral parts of katepisternum blackish. Anepisternum with shallow sulcus; golden yellow setulose anterodorsally and posterodorsally, widely bare medially and ventrally. Anepimeron golden yellow setulose. Katepisternum with patch of white setulae dorsally and a few white setulae ventrally. Katatergite long microtrichose, anatergite short microtrichose. Calypter and halter pale yellow.

Wing: hyaline; microtrichose, except bare on basal 1/4 of cell r_1_, most of cell br (only microtrichose along vena spuria), posterobasal 1/2 of cell bm, anterobasal 1/3 of cell cup, and basomedian 3/5 of alula.

Legs: femora black; yellow to white setulose. Tibiae black, apices narrowly brown; white setulose. Tarsi black with apical tarsomere yellowish brown; black setulose dorsally, yellow setulose ventrally. Coxae and trochanters blackish; yellow and white setulose.

***Abdomen*.** Tergites blackish, with lateral and posterior margins brown. Tergite 1 yellowish white setulose. Tergite 2 shiny with three large dull maculae; yellowish white setulose. Tergite 3 dull on most of surface, with lateral and posterior margins shiny; yellowish white setulose on shiny parts, black setulose on dull parts. Tergite 4 semi-shiny; reddish yellow setulose laterally and posteriorly, black setulose anteriorly and medially, white setulose in anterolateral corners. Sternites blackish; yellow setulose. Genitalia as in Fig. 141.

**Female.** As male, except for following differences. Face, frons, and vertex black setulose except for pair of small patches of white setulae along eye margin. Scutum, scutellum, and pleura black setulose. Tergite 5 yellow to yellowish brown; yellow setulose.

##### Diagnosis.

Body length: male 9–10 mm (*n* = 10), female 11–12 mm (*n* = 2). Superficially, this species looks most similar to *S.boti* Reemer, sp. nov., *S.chloraspis* Reemer, sp. nov., and *S.pallitarsis* Reemer & Mengual, sp. nov., which are of similar size and colouration. These four species all have a black face, entirely dark tibiae, a uniformly coloured postpedicel, and a large rectangular dull area on tergite 3. The combination of the following characters distinguishes *S.varicaudata* Reemer & Mengual, sp. nov. from the other three species: wing cell dm entirely microtrichose (partly bare in *S.boti* Reemer, sp. nov.), scutum and scutellum without strong metallic green shine (green metallic in *S.chloraspis* Reemer, sp. nov.), tergite 2 with lateral dull parts large (Fig. 33) (small in *S.boti* Reemer, sp. nov. and *S.pallitarsis* Reemer & Mengual, sp. nov.; Figs 30, 32), hind metatarsus dorsally black (orange in *S.boti* Reemer, sp. nov. and *S.pallitarsis* Reemer & Mengual, sp. nov.). Male genitalia as in Fig. 141.

##### Notes.

In the studied male specimens, the degree to which tergite 4 is covered with red setulae varies from only the apical 1/3 to the entire tergite (Figs 123–125). The colour of the setulae on the vertex also varies from entirely golden yellow to entirely black.

##### Etymology.

The specific epithet is composed of the Latin words *varius* (different) and *cauda* (tail). The name refers to the variability of the colouration of both integument and setulosity of tergite 4.

##### Molecular data.

Several DNA barcodes were successfully obtained from multiple specimens from Ecuador and they were recovered in a well-supported cluster (BS = 98.8) in the NJ tree. The intraspecific similarity ranges from 99.111 to 100%.

##### Distribution.

This species is only known from Ecuador, where it was found on eastern and western slopes of the Andes at elevations between 1800 and 2200 meters.

#### ﻿ Serichlamys vexilliphallus

Taxon classificationAnimaliaDipteraSyrphidae

Reemer & Mengual sp. nov.

C4D2B1C6-D080-536F-8F69-0EB346C985E3

https://zoobank.org/003A045E-AF34-4056-98A0-7B93D0870B87

Figs 7 , 126–130 , 150–153

##### Type material.

***Holotype*.** Ecuador • 1 ♂, holotype of *Serichlamysvexilliphallus* sp. nov.; Napo Prov., Huahua Sumaco, km 44 on Hollin-Loreto road; 17 Dec. 1989; M.J. Wasbauer & H. Real leg.; CSCA.

Label 1: “ECUADOR: Napo Prov. // Huahua Sumaco, km 44 // on Hollin-Loreto rd // XII-17-1989 mal. trap // M. & J. Wasbauer, H. Real”; label 2: “Serichlamys sp. nov. // Det. M. Reemer 2022 /./ Specimen code MR1480”. ***Paratypes*.** Ecuador • 1 ♂; Napo Prov., Huahua Sumaco, km 45 on Hollin-Loreto road; 14 Dec. 1989; M.J. Wasbauer & H. Real leg.; CSCA • 2 ♂; Napo Prov., Huahua Sumaco, km 45 on Hollin-Loreto road; 15 Dec. 1989; M.J. Wasbauer & H. Real leg.; CSCA • 2 ♂; Napo Prov., Huahua Sumaco, km 45 on Hollin-Loreto road; 16 Dec. 1989; M.J. Wasbauer & H. Real leg.; CSCA • 1 ♂; Napo Prov., Huahua Sumaco, km 45 on Hollin-Loreto road; 18 Dec. 1989; M.J. Wasbauer & H. Real leg.; RMNH [specimen code MR1483] • 2 ♂; Napo Prov., Huahua Sumaco, km 45 on Hollin-Loreto road; 20 Dec. 1989; M.J. Wasbauer & H. Real leg.; CSCA • 1 ♂; Napo Prov., Huahua Sumaco, km 45 on Hollin-Loreto road; 21 Dec. 1989; M.J. Wasbauer & H. Real leg.; RMNH• 2 ♂; Morona Santiago: Miazal, 50 km SE Macas; 300 m asl; 4–7 Jan. 1993; M. & J. Wasbauer leg.; CSCA • 5 ♂; Napo, Misahualli nr. Tena; 3–8 Oct. 1999; S.R. Keller leg.; LACM • 1 ♂; Napo Prov., Jatun Sacha Biological Station; 01°03'58.4"S, 77°37'0.6"W; 26 Aug. 2019; X. Mengual leg; ZFMK [ZFMK-DIP-00068685]; GenBank accession no. PQ629008.

**Figures 126–130.**
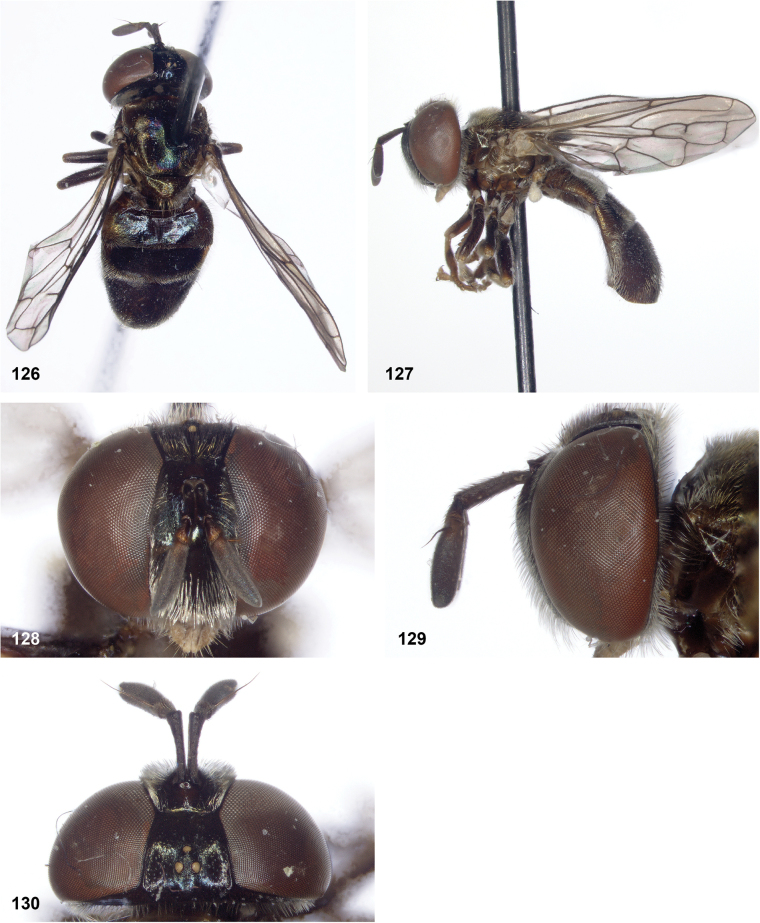
*Serichlamysvexilliphallus* Reemer & Mengual, sp. nov. male, holotype **126** habitus, dorsal **127** habitus, lateral **128** head, frontal **129** head, lateral **130** head, dorsal.

##### Additional specimens.

Panama • 1 ♂; Chiriqui province, 8 km S Boquete; 08°41'54"N, 82°27'06"W; 800 m asl; 16–30 Jun. 2012; F.D. Parker & T.D. McIntyre leg.; CSCA. PERU • 1 ♂; Amazonas, Distr. Aguas Verdes, Bagua/Tarapoto Rd. (5N) at km 403; 5°41'23"S, 77°38 13"W; 1125 m asl; 15–22 May 2009; M.E. Irwin & G. A. Amaya leg; CNC [J. Skevington molecular specimen #9948] • 1 ♂; SAM: around San Roque de Cumbaza; 6°23'4.96"S, 76°25'53"W; 15–31 Jan. 2015; T. Faasen leg; RMNH [M. Reemer specimen code MR0565; also CNC464838]; GenBank accession no. PQ629015 • 1 ♀; Fundo Chela; 4 Jun. 1964; 1100 m asl; J. Schunke leg.; NHMUK. Colombia • 1 ♂; Putumayo, Villa Garzon, 8 mi. S. Mocoa; 3 Aug. 1978; M. Cooper leg.; NHMUK [NHMUK013624759] • 1 ♂; Putumayo, Villa Garzon; 400 m asl; 30 Aug. 1984; M. Cooper leg.; NHMUK [NHMUK013624761]; • 1 ♂; Putumayo, Villa Garzon; 400 m asl; 7 Oct. 1984; M. Cooper leg.; NHMUK [NHMUK013624743]; 1 ♂; Putumayo, Villa Garzon; 400–600 m asl; 31 Dec. 1990; M. Cooper leg.; NHMUK [NHMUK013624760] • 1 ♂; Caqueta, Yuruyaco, 73 km SW Florencia; 5 Feb. 1979; M. Cooper leg.; NHMUK [NHMUK013624748]. ECUADOR • 1 ♀; Morona-Santiago, Sucua [?]; 850 m asl; 15 Jul. 194; M. Cooper leg.; NHMUK [NHMUK013624762] • 1 ♂; Morona-Santiago, Cord de Cutucu, 6 km E. of Macas; 1100 m asl.; 17 Mat 1987; M. Cooper leg.; NHMUK [NHMUK013624746] • 1 ♀; Morona-Santiago, Cord de Cutucu, 6 km E. of Macas; 1100 m asl.; 17 Mat 1987; M. Cooper leg.; NHMUK [NHMUK013624745] • 1 ♂; Napo, Muyuna, 5 km W of Tena; 500 m asl.; 13 Apr. 1981; leg. M. Cooper; NHMUK [NHMUK013624749] • 1 ♂; Napo, near Myuna; [no date on label]; M. Cooper leg.; NHMUK [NHMUK013624832].

##### Description

**(based on holotype). Adult male** Body size: 6.5 mm.

***Head*.** Face occupying slightly < 1/3 of head width in frontal view; shiny black with faint green hue; white setulose. Gena very narrow; black; white setulose. Oral margin laterally not produced. Frons black; medially bare, laterally with mixed white and black setulae. Vertex black; black setulose except yellowish setulose along anterior and posterior margins. Occiput black; dorsal 1/3 pale yellow setulose, otherwise white setulose. Eye bare. Antennal fossa slightly higher than wide. Antenna blackish brown; antennal ratio ~ 4:1:4.5.

***Thorax*.** Scutum shiny black, with green sheen on median 1/3 and bronze sheen on lateral 1/3; black golden yellow setulose on anterior 1/4 and narrowly along lateral and posterior margins, otherwise black setulose. Postpronotum and postalar callus yellowish brown; yellow setulose. Scutellum trapezoid, with two calcars as long as ~ 1/4 of scutellar length; shiny dark brown with faint greenish sheen, except calcars yellow; yellow setulose. Pleura shiny dark to yellowish brown. Anepisternum with shallow sulcus; yellow setulose anterodorsally and posterodorsally, widely bare medially and ventrally. Anepimeron yellow setulose with a few black setulae dorsally. Katepisternum with patch of white setulae dorsally and small patch of white setulae ventrally. Katatergite long microtrichose, anatergite short microtrichose. Calypter pale yellow, halter white.

Wing: hyaline; microtrichose, except bare on basal cell bc, posterobasal 1/3 of cell c, basal 3/5 of cell r_1_, basal 1/8 of cell r_2+3_, most of cell br (only microtrichose along vena spuria), slightly more than posterior 1/2 of cell bm, anterior 1/2 of cell cup, and basomedian 2/3 of alula.

Legs: femora blackish brown with yellow apices; yellow setulose. Tibiae pale yellow at basal 2/3, pale brown at apical 1/3, and blackish brown around cicatrix; yellow setulose except black setulose on inner side at apical 1/3. Tarsi pale brown; black setulose dorsally, yellow setulose laterally and ventrally. Coxae and trochanters brown; pale yellow to white setulose.

***Abdomen*.** Tergites blackish brown, mostly shiny except for large part of tergite 3. Tergite 1 white setulose. Tergite 2 shiny except for small median dull macula, with distinct metallic sheen at lateral 1/4; whitish setulose, except for some black setulae along lateral margin. Tergite 3 dull black, except for narrow fascia with bronze sheen along posterior and lateral margins; dull part short black setulose except for pale setulae posteromedially, shiny parts longer yellowish white setulose. Tergite 4 entirely shiny but not metallic; mostly short black setulose, but longer pale yellow to whitish setulose along lateral margins and on pair of large posteromedian patches. Sternites blackish brown; all sternites white setulose. Genitalia as in Figs 150–153: phallus with large, subapical flag-like projection.

**Female (based on paratype)** As male, except for usual sexual dimorphism and following differences. Scutum largely black setulose, except for yellowish setulae along anterior margin. Tergites somewhat paler brown than in male.

##### Diagnosis.

Body length: male 6.0–7.0 mm (*n* = 19), female 8.5–9 mm (*n* = 3). The combination of a black face with a contrasting colour pattern on the tibiae (Fig. 7) sets this species apart from all other species in the genus. Besides, the male genitalia are very distinct in the fact that the phallus carries a ‘flag-like’ apicodorsal projection (Figs 150–153).

##### Notes.

This is the only known species of *Serichlamys* in which the male has a flag-like appendix on the phallus. Some slight variation occurs in the shape of this appendix (Figs 150–153), but this is considered to be intraspecific. Little variation among the specimens (mostly subtle differences in colouration) was noted in external characters, except that the colour of setulosity on the scutum may vary between entirely golden yellow to mostly black. No habitat information is indicated on the labels of the specimens, but the localities suggest that they were found at the margins of tropical rain forest.

##### Etymology.

The specific epithet is a noun in apposition composed of the Latin words *vexillum* (flag) and *phallus* (penis). It refers to the characteristic dorsal projection on the phallus in males of this species.

##### Molecular data.

A specimen from Ecuador and another from Peru were sequenced. Both DNA barcodes cluster together with high-support (BS = 100) and they have a similarity of 97.833%.

##### Distribution.

This species is known from Ecuador (*n* = 13), Peru (*n* = 1) and Panama (*n* = 1). In the first two countries, it has been found at elevations between 300 and 1100 meter, at slopes of the Andes and in lowland rainforest. In Panama it was found in rainforest at 800 meters of altitude. Based on these records, it can be expected that this species also occurs in Colombia.

#### ﻿ Serichlamys xanthocnemia

Taxon classificationAnimaliaDipteraSyrphidae

Reemer sp. nov.

FA373097-7E96-5080-A27F-EC03D05F5E69

https://zoobank.org/7D3E2153-4EA4-43CA-9760-2D6375C3984A

Figs 38 , 131–137 , 154

##### Type material.

***Holotype*.** Brazil • 1 ♂, holotype of *Serichlamysxanthocnemia* sp. nov.; São Paulo, Peruibe; 4 Aug. 1993; O. Niehuis leg.; RMNH.

##### Description

**(based on holotype). Adult male** Body size: 7 mm.

***Head*.** Face occupying ~ 1/3 of head width in frontal view; shiny yellowish brown; white setulose, except for bare median part. Gena yellow; white setulose. Oral margin laterally hardly produced. Frons dark brown; medially bare, laterally black setulose. Vertex brown; golden yellow setulose anteriorly and posteriorly, black setulose in between. Occiput blackish; white setulose. Eye bare. Antenna: scape and pedicel black, postpedicel dark brown; antennal ratio ~ 3.5:1:4.5.

**Figures 131–137.**
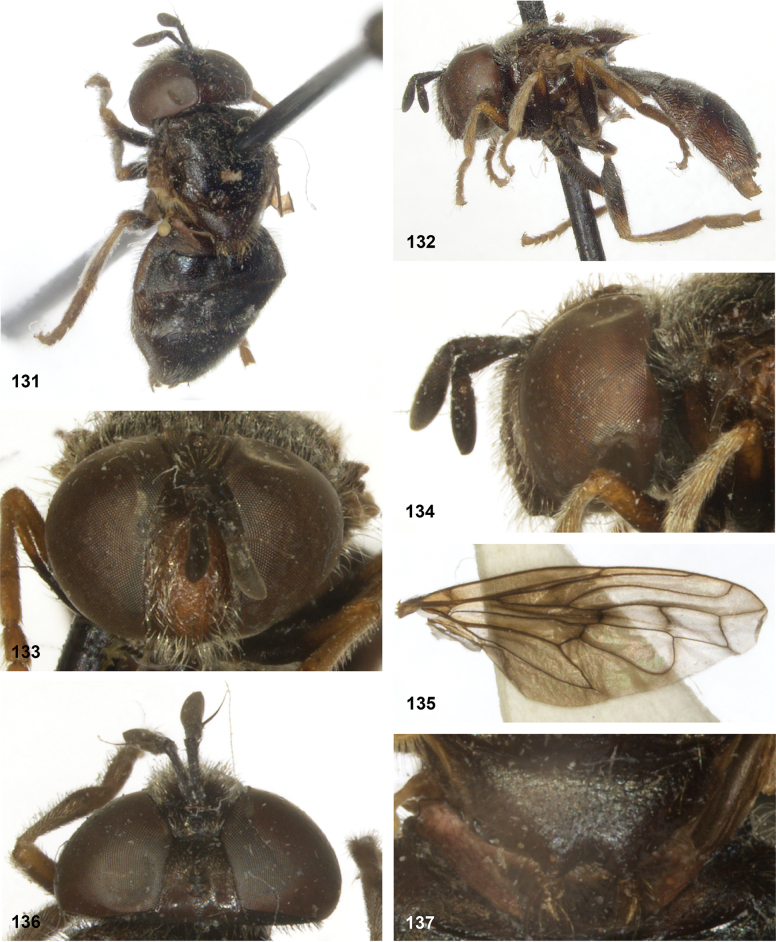
*Serichlamysxanthocnemia* Reemer, sp. nov. male, holotype **131** habitus, dorsal **132** habitus, lateral **133** head, frontal **134** head, lateral **135** head, dorsal **136** wing **137** scutellum.

***Thorax*.** Scutum blackish with bronze sheen, margins brown; golden yellow setulose. Postpronotum and postalar callus brown; golden yellow setulose. Scutellum trapezoid, brown; black setulose medially, golden yellow setulose laterally and posteriorly; with two pale, acute, converging calcars as long as ~ 1/3 of scutellar length. Pleura blackish brown, except katepimeron and margins of some other pleurites yellowish brown. Anepisternum with shallow sulcus; yellowish white setulose anterodorsally and posterodorsally, widely bare medially and ventrally. Anepimeron yellowish white setulose. Katepisternum with patch of white setulae dorsally and a few white setulae ventrally. Katatergite long microtrichose, anatergite short microtrichose. Calypter and halter yellowish white.

**Figures 138–141.**
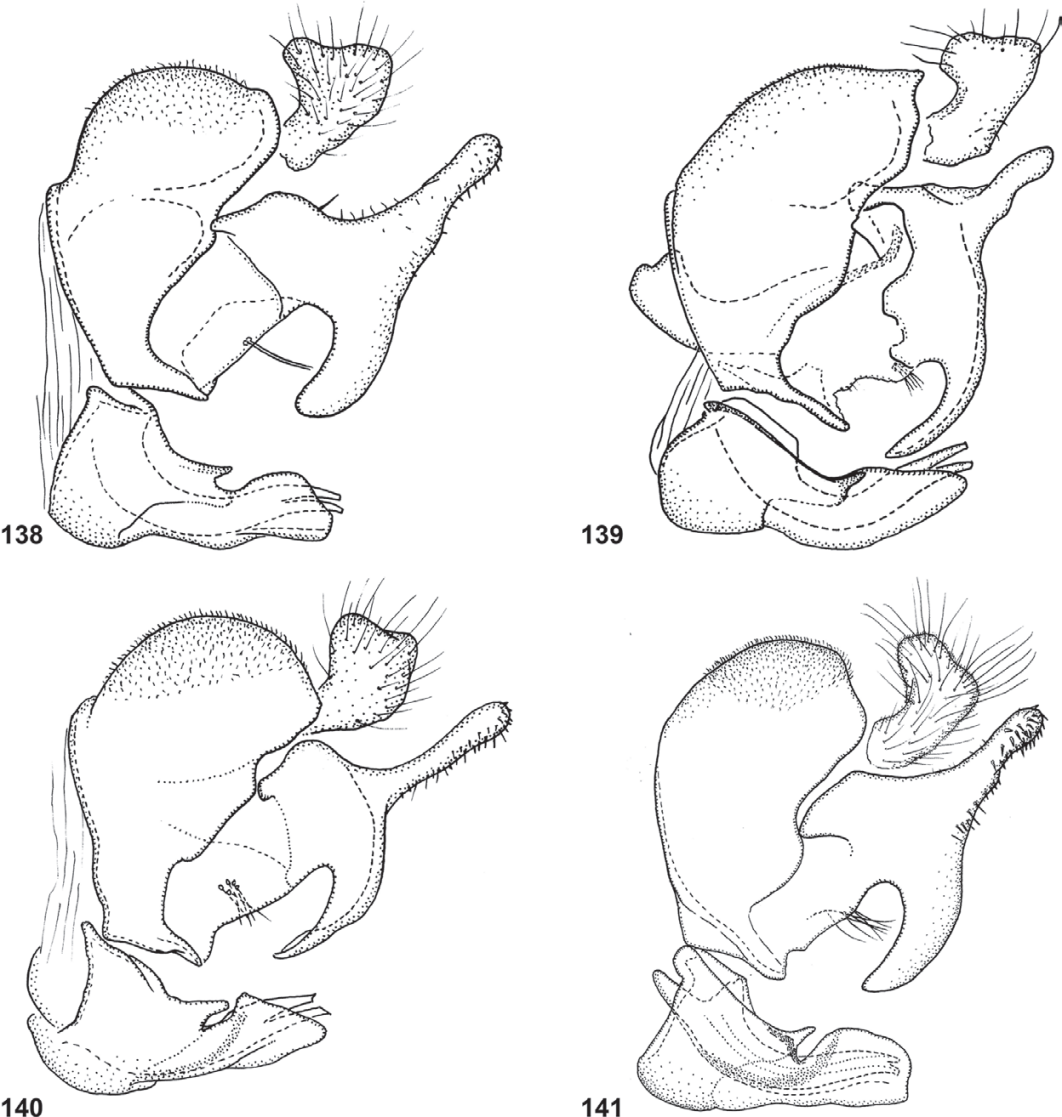
*Serichlamys* male genitalia **138***S.boti* Reemer, sp. nov. holotype **139***S.chloraspis* Reemer, sp. nov. paratype **140***S.pallitarsis* Reemer & Mengual, sp. nov. paratype, coll. USNM, specimen code MR1595 **141***S.varicaudata* Reemer & Mengual, sp. nov., coll. ZFMK, specimen code MR425.

Wing: hyaline; microtrichose, except bare on basal 2/3 of cell br (and microtrichose along vena spuria), basal 1/3 of cell bm, and anterobasal 1/10 of cup.

**Figures 142–145.**
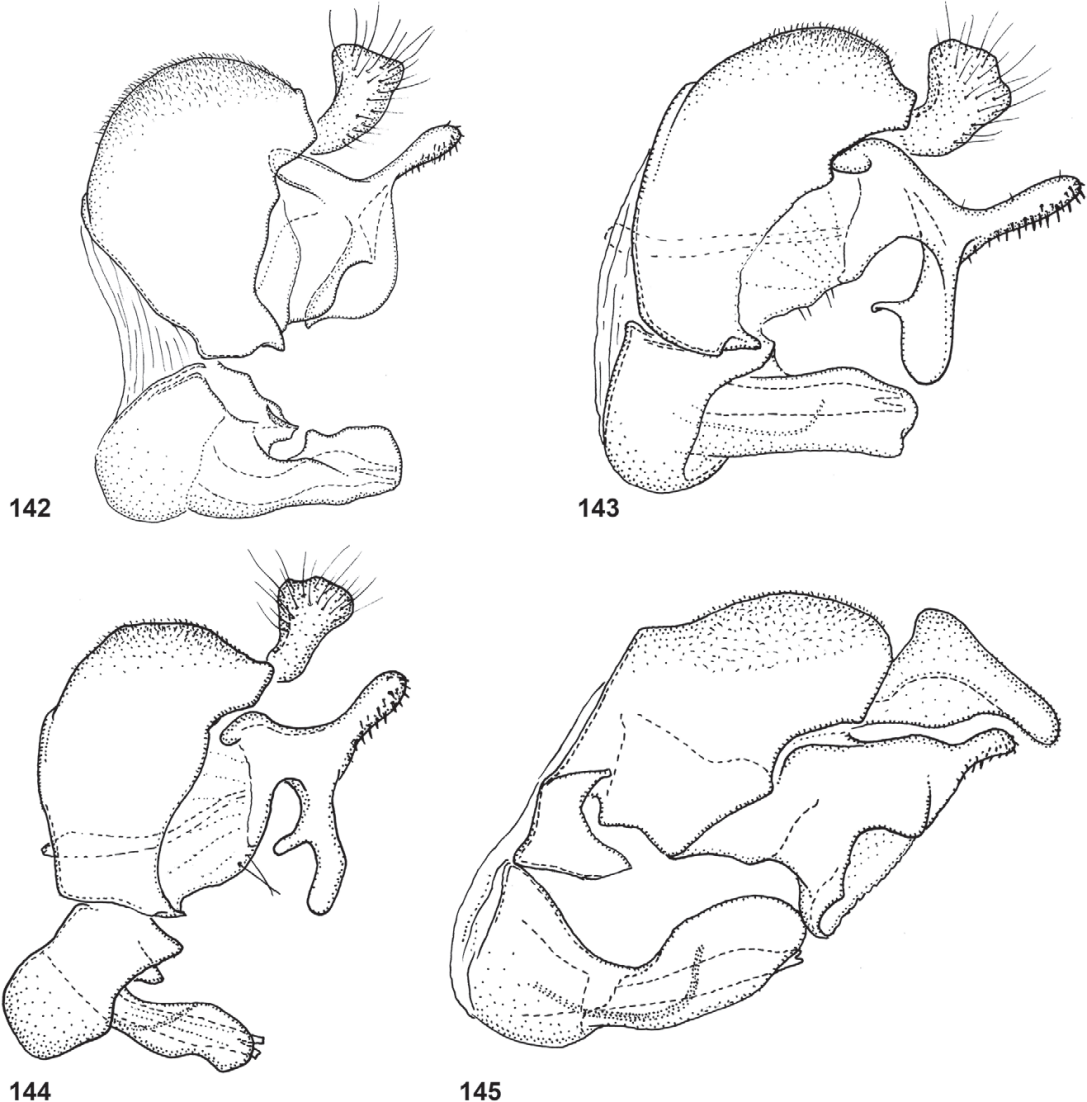
*Serichlamys* male genitalia **142***S.melamitis* Reemer, sp. nov. paratype, coll. MZUSP, specimen code MR1591 **143***S.mellimitis* Reemer, sp. nov. holotype **144***S.mitis* (Curran, 1940) coll. RMNH, specimen code MR172 **145***S.trigonoides* Reemer, sp. nov. holotype.

Legs: yellow, except basal 1/2 of fore femur and basal 2/3 of mid and hind femora black; pale yellow setulose, except femora largely black setulose. Coxae and trochanters blackish brown; yellow to white setulose.

**Figures 146–149.**
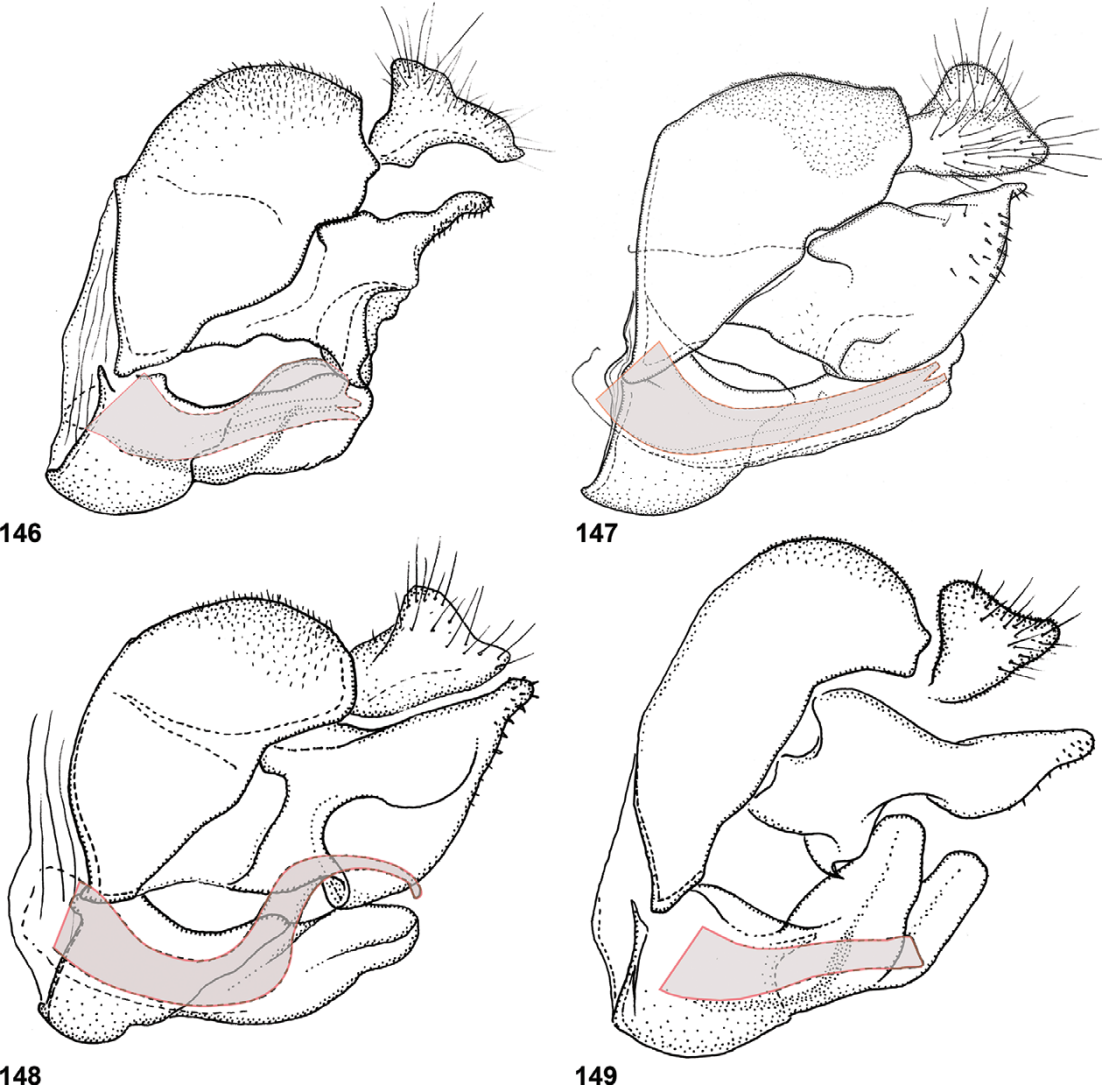
*Serichlamys* male genitalia (phallus grey-shaded) **146***S.mus* (Curran, 1936) coll. USNM, USNMENT01866933**147***S.spathulata* Reemer, sp. nov. coll. JTS, specimen code MR598 **148***S.serpentiphallus* Reemer, sp. nov. coll. RMNH, specimen code MR381 **149***S.simpliciphallus* Reemer, sp. nov. holotype.

***Abdomen*.** Tergites blackish, except lateral margins brown (brown parts widening towards apex of abdomen). Tergite 1 white setulose. Tergite 2 white setulose. Tergite 3 dull on most of surface, with lateral and posterior margins shiny; black setulose on dull parts, white setulose on shiny parts. Tergite 4 shiny; yellowish setulose laterally and posteriorly, black setulose anteriorly and medially. Sternites yellowish brown; yellowish setulose with some black setulae on sternite 4. Genitalia as in Fig. 154.

**Figures 150–154.**
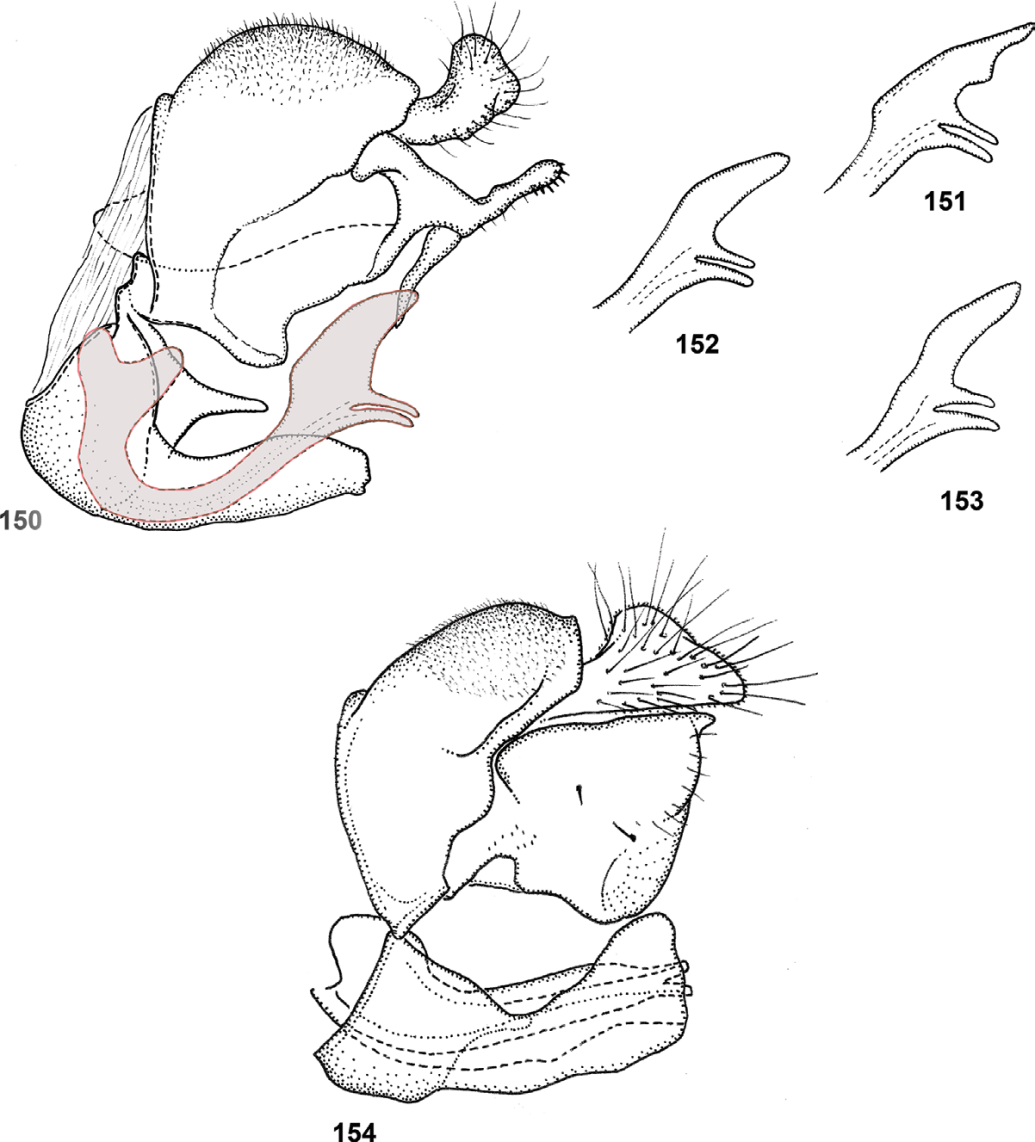
*Serichlamysvexilliphallus* Reemer & Mengual, sp. nov. male genitalia, with variations in shape of distal part of phallus **150** holotype (Ecuador) **151** paratype, specimen code MR1483 (Ecuador, coll. RMNH) **152** specimen code MR1482 (Panama, coll. CSCA) **153** specimen code MR565 (Peru, coll. RMNH) **154***Serichlamysxanthocnemia* Reemer, sp. nov. male, holotype, genitalia.

**Female.** Unknown.

##### Diagnosis.

Body length: male 7 mm (*n* = 1). This is the only known species of *Serichlamys* which combines a yellow face, femora which are 2/3 black, and yellow tibiae and tarsi. Male genitalia as in Fig. 154. Female unknown.

##### Etymology.

The species name is derived from the Greek words *xanthos* (yellow) and *kneme* (tibia). This name was chosen because of the yellow tibiae of this species.

##### Distribution.

This species is only known from the Brazilian state São Paulo.

### ﻿Neotropical distribution of *Serichlamys*

**Figure 155.**
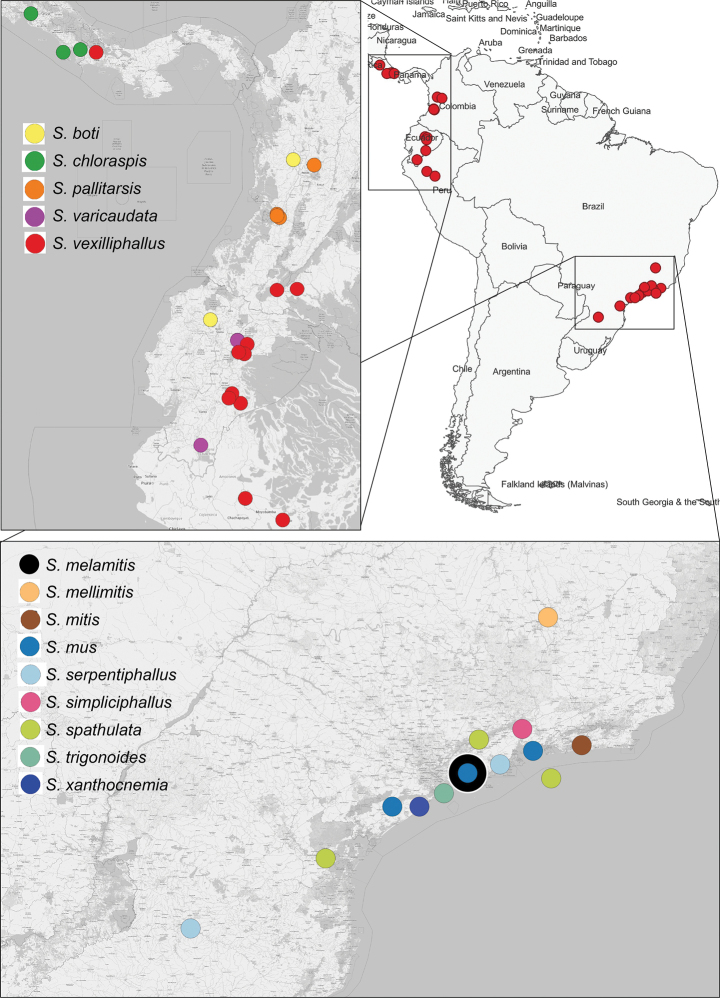
Distribution of *Serichlamys* species in the Neotropical region.

The distribution of the genus *Serichlamys* in the Neotropical region appears to be disjunct (Fig. 155). One group of species (*S.boti* Reemer, sp. nov., *S.chloraspis* Reemer, sp. nov., *S.pallitarsis* Reemer & Mengual, sp. nov., *S.varicaudata* Reemer & Mengual, sp. nov., and *S.vexilliphallus* Reemer & Mengual, sp. nov.) occurs at mid and low elevations on and around the slopes of the Andes in Colombia, Ecuador and Peru, with northern extensions into Panama (*S.vexilliphallus* Reemer & Mengual, sp. nov.) and Costa Rica (*S.chloraspis* Reemer, sp. nov.). The other group of species (including the nine remaining known species) occurs in the south-eastern states of Brazil: Minas Gerais, Rio de Janeiro, São Paulo, Paraná, and Santa Catarina.

### ﻿DNA barcoding

**Figure 156.**
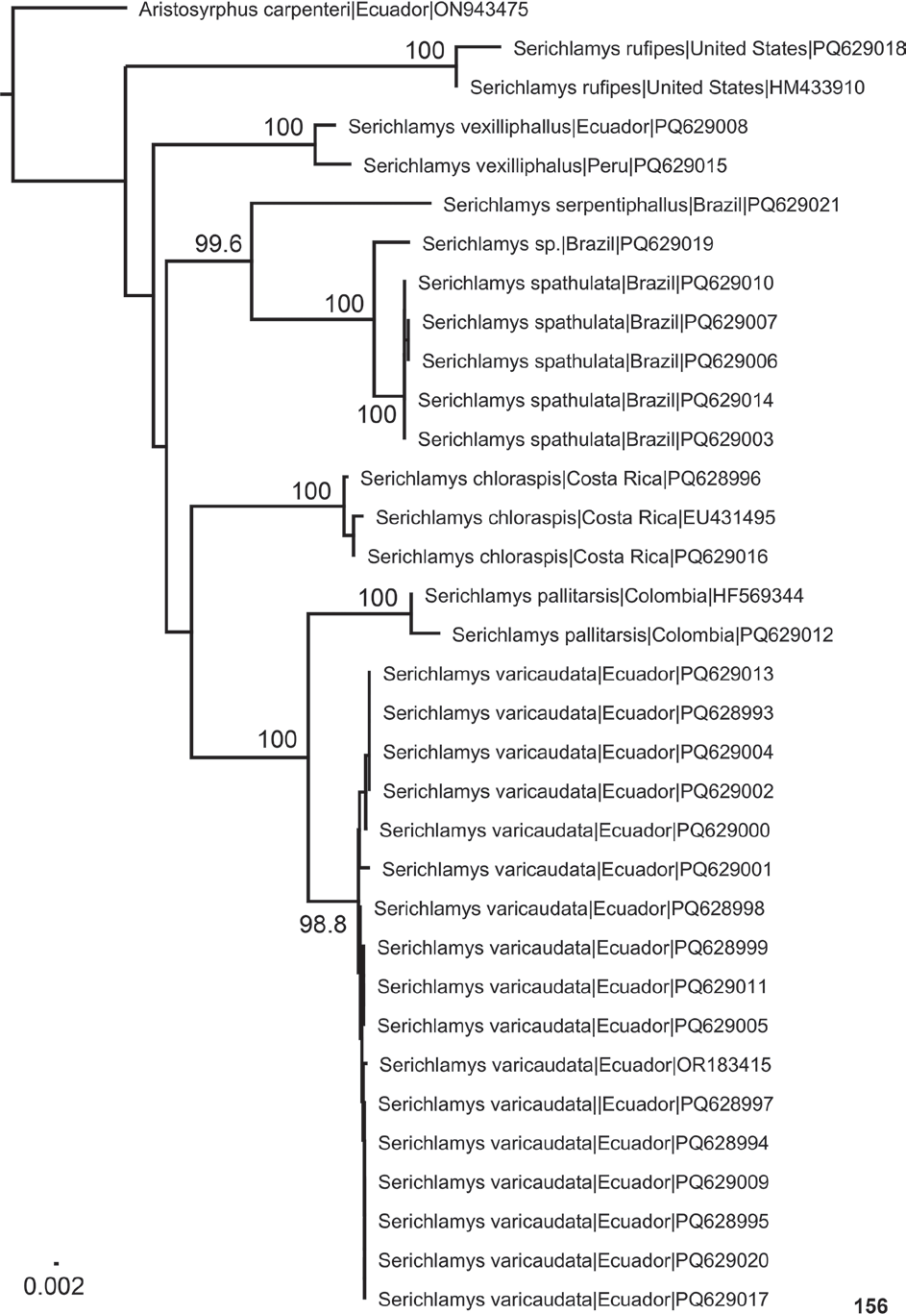
Neighbor-Joining tree using Jukes-Cantor model based on COI sequences of *Serichlamys*, with *Aristosyrphuscarpenteri* (Hull, 1945) constrained as the outgroup. Bootstrap support values (>80%) are indicated at the nodes. The name for each specimen has: the name of the species | country of origin | GenBank accession number.

We were able to download five publicly available COI sequences of *Serichlamys* and we newly sequenced 28 specimens (Fig. 156). All taxa with molecular data were clustered with high support (BS > 98) following the morphological identifications. Overall, the intraspecific uncorrected distances were < 0.01 or 1%, ranging from 0 to 0.00889 (equivalent to an intraspecific similarity of 99.111–100%), with the exception of the two sequences of *S.vexilliphallus* Reemer & Mengual, sp. nov., which differ 0.02167 (similarity = 97.833%).

The interspecific uncorrected distance is generally higher, with values ranging from 0.02584–0.02736 between *Serichlamysspathulata* Reemer, sp. nov. and *Serichlamys* sp. (CNC1059093) to > 0.16462 between *S.rufipes* and other *Serichlamys* species, and usually with a distance > 0.05 between species pairs, except for the already mentioned pair *S.spathulata* Reemer, sp. nov. and *Serichlamys* sp. (CNC1059093).

## ﻿Discussion

In comparison with many other genera of Microdontinae, *Serichlamys* displays a remarkable variation in the structure of the male genitalia. Especially noteworthy are the differences in the shape of the phallus between *Serichlamysmus*, *S.serpentiphallus* Reemer, sp. nov., *S.simpliciphallus* Reemer, sp. nov. and *S.spathulata* Reemer, sp. nov. (Figs 146–149), four species which are very similar in external characters. Usually, in Microdontinae, the shape of the phallus is very similar between species of the same genus, for instance see *Peradon* Reemer, 2013 (Reemer et al. 2019) or *Stipomorpha* Hull, 1945 (Reemer 2013b). As nothing is known about the mating biology of Microdontinae in general and *Serichlamys* in particular, one can only speculate about the explanation for this variation.

## Supplementary Material

XML Treatment for Serichlamys boti

XML Treatment for Serichlamys chloraspis

XML Treatment for Serichlamys melamitis

XML Treatment for Serichlamys mellimitis

XML Treatment for Serichlamys mitis

XML Treatment for Serichlamys mus

XML Treatment for Serichlamys pallitarsis

XML Treatment for Serichlamys serpentiphallus

XML Treatment for Serichlamys simpliciphallus

XML Treatment for Serichlamys spathulata

XML Treatment for Serichlamys trigonoides

XML Treatment for Serichlamys varicaudata

XML Treatment for Serichlamys vexilliphallus

XML Treatment for Serichlamys xanthocnemia

## Footnotes

The following species are very similar in morphology and colouration. The most reliable characters are found in the male genitalia. All other characters are to be used with great caution.

## ﻿Additional information

### Author ORCIDs

Ximo Mengual https://orcid.org/0000-0002-6185-9404

### Data availability

All of the data that support the findings of this study are available in the main text or Supplementary Information.

## Supplementary materials

Supplementary material 1 Matrix of Neighbour-Joining distances between COI sequences of *Serichlamys* specimens Data type xlsx https://binary.pensoft.net/file/1359661This dataset is made available under the Open Database License (http://opendatacommons.org/licenses/odbl/1.0/). The Open Database License (ODbL) is a license agreement intended to allow users to freely share, modify, and use this Dataset while maintaining this same freedom for others, provided that the original source and author(s) are credited.Menno Reemer, Ximo Mengual
